# Emerging early diagnostic methods for acute kidney injury

**DOI:** 10.7150/thno.71064

**Published:** 2022-03-21

**Authors:** Zuoxiu Xiao, Qiong Huang, Yuqi Yang, Min Liu, Qiaohui Chen, Jia Huang, Yuting Xiang, Xingyu Long, Tianjiao Zhao, Xiaoyuan Wang, Xiaoyu Zhu, Shiqi Tu, Kelong Ai

**Affiliations:** 1 Xiangya School of Pharmaceutical Sciences, Central South University, Changsha, Hunan, P.R. China, 410078.; 2 Hunan Provincial Key Laboratory of Cardiovascular Research, Xiangya School of Pharmaceutical Sciences, Central South University, Changsha, 410078, China.; 3 Department of Pharmacy, Xiangya Hospital, Central South University, Changsha, Hunan, P.R. China, 410008.; 4 National Clinical Research Center for Geriatric Disorders, Xiangya Hospital, Central South University, Changsha, Hunan, P.R. China, 410008.; 5 Hunan Key Laboratory of Oral Health Research, Hunan 3D Printing Engineering Research Center of Oral Care, Hunan Clinical Research Center of Oral Major Diseases and Oral Health, Xiangya Stomatological Hospital, and Xiangya School of Stomatology, Central South University, Hunan, 410008, Changsha, China.

**Keywords:** Acute kidney injury, Machine learning, Reactive oxygen species and nitrogen species, Neutrophil gelatinase-associated lipocalin, kidney injury molecule-1, γ-glutamyl transpeptidase, miRNA-21, Early diagnosis.

## Abstract

Many factors such as trauma and COVID-19 cause acute kidney injury (AKI). Late AKI have a very high incidence and mortality rate. Early diagnosis of AKI provides a critical therapeutic time window for AKI treatment to prevent progression to chronic renal failure. However, the current clinical detection based on creatinine and urine output isn't effective in diagnosing early AKI. In recent years, the early diagnosis of AKI has made great progress with the advancement of information technology, nanotechnology, and biomedicine. These emerging methods are mainly divided into two aspects: First, predicting AKI through models construct by machine learning; Second, early diagnosis of AKI through detection of newly-discovered early biomarkers. Currently, these methods have shown great potential and become an attractive tool for the early diagnosis of AKI. Therefore, it is very important to discuss and summarize these methods for the early diagnosis of AKI. In this review, we first systematically summarize the application of machine learning in AKI prediction algorithms and specific scenarios. In addition, we introduce the key role of early biomarkers in the progress of AKI, and then comprehensively summarize the application of emerging detection technologies for early AKI. Finally, we discuss current challenges and prospects of machine learning and biomarker detection. The review is expected to provide new insights for early diagnosis of AKI, and provided important inspiration for the design of early diagnosis of other major diseases.

## 1. Introduction

Acute kidney injury (AKI) is a common clinical syndrome characterized by a sudden dysfunction of the kidney. The kidney is an important metabolic organ of the human body. Many factors can cause AKI, including heart failure, sepsis, hemorrhage, nephrotoxic drugs, and COVID-19 *etc.*^1^-^8^. For example, Hirsh *et al.* reported the high prevalence among hospitalized patients with COVID-19 (36.6%) 9. It is estimated that 20% of hospitalized patients deteriorate to AKI, and 10% of AKI patients require renal replacement therapy (RRT). The mortality rate of patients requiring RRT is as high as 50%. Patients recovering from AKI have a higher risk of chronic kidney disease and even end-stage renal disease 10-13. The treatment options for AKI are very limited. Early diagnosis of AKI and taking appropriate preventive measures can effectively recover from AKI. Currently, the diagnosis standard of AKI is based on the level of serum creatinine (SCR) and urine output (UO) according to the guidelines issued by Kidney Disease: Improving Global Outcomes in 2012 14. However, SCR and UO are non-specific and delayed for the early diagnosis of AKI. SCR can be affected by many non-renal factors. For example, individuals with normal renal function may experience the rise of SCR due to high muscle mass or intake of certain drugs (such as trimethoprim and cimetidine) 15. In addition, only persistent oliguria is an effective signal of acute kidney injury. In this case, UO cannot diagnose AKI in time 16. Moreover, the changes of SCR and UO have a long-time delay compared with the important structural changes of the kidney of AKI. When SCR and UO change significantly, renal function has been severely impaired, which easily makes AKI treatment miss the best time of intervention 17, 18.

Imaging diagnosis includes ultrasound, computed tomography *etc.* can assess kidney morphology and provide insights into the function, perfusion, and possible etiology for AKI 19, 20. However, these methods have disadvantages such as low resolution, radiation damage, and the potential risk of increased nephrotoxicity caused by contrast agents, and is not suitable for the diagnosis of early AKI.

**Figure 1:**
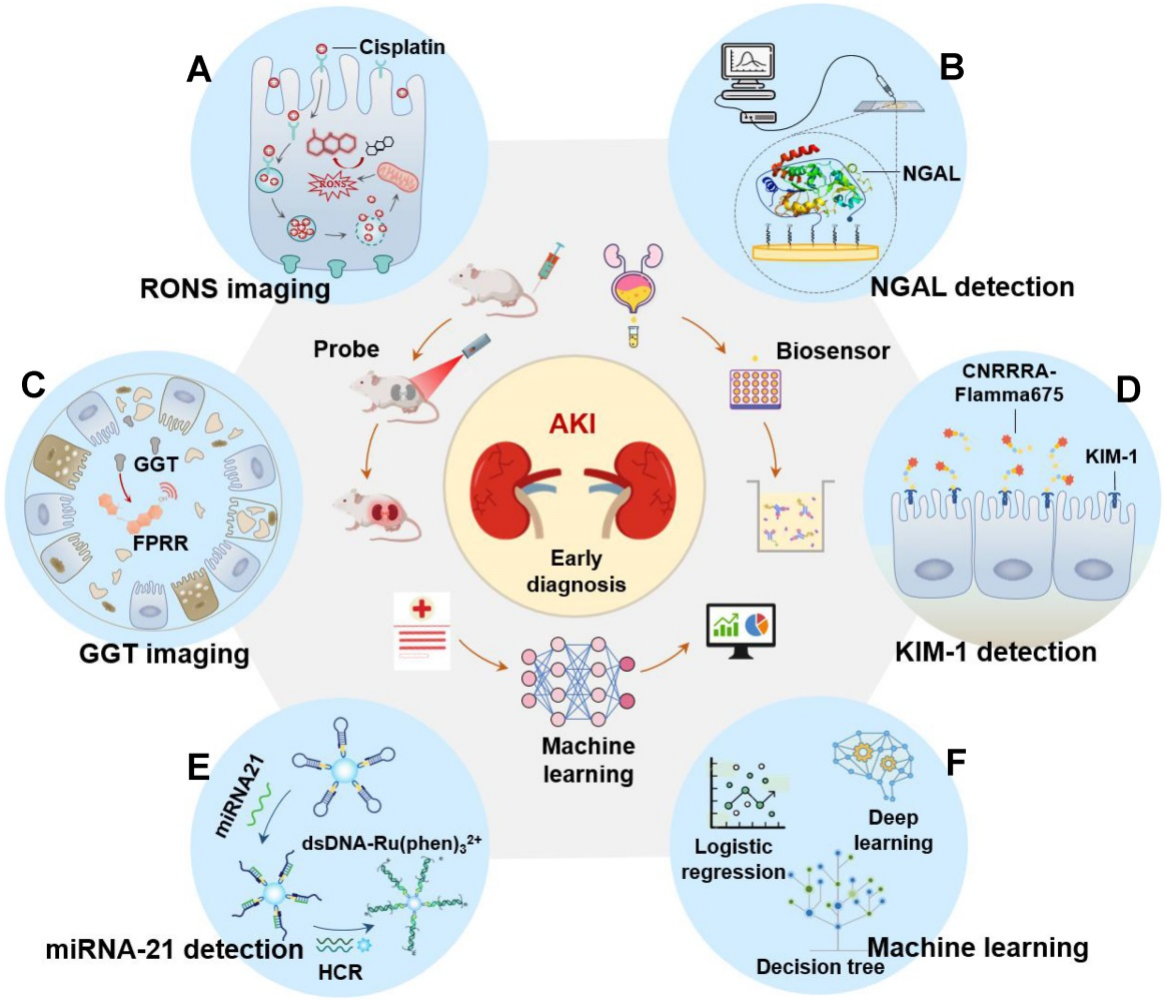
The latest applications of early diagnosis of AKI fall into three categories: optical probes imaging, biosensors and machine learning prediction models. The detected biomarkers involved are (A) RONS, (B) NGAL, (C) GGT, (D) KIM-1, and (E) miRNA21. (F) The algorithms involved in machine learning are logistic regression, deep learning, decision tree and so on.

In recent years, the early diagnosis of AKI has made great progress with the advancement of information technology, nanotechnology, and biomedicine 21, 22. On the one hand, new functions of artificial intelligence in biomedicine are constantly being discovered 23-25. Machine learning, as a branch of artificial intelligence, can effectively predict AKI by building predictive models based on analyzing large data sets related to medical treatments and outcomes 26. On the other hand, more and more effective and potential early biomarkers have been discovered with the in-depth study of AKI pathology, such as neutrophil gelatinase associated lipoprotein (NGAL), γ-glutamyl transpeptidase (GGT), kidney injury molecule-1 (KIM-1), microRNA (miRNA) and excess reactive oxygen species and nitrogen (RONS) 27-30. The concentration of these biomarkers in the kidney or body fluid (like blood or urine) is significantly increased before the onset of renal organic and functional diseases. Therefore, these biomarkers are more sensitive for early AKI than SCR and UO. However, as clinical needs expand, traditional detection methods (such as ELISA and PCR) for these new biomarkers are no longer applicable. On this basis, many kinds of biosensor (like optical probes, electrochemical probes, and surface plasmon resonance (SPR) probes) based on advanced nanotechnology, deoxyribonucleic acid (DNA) technology, and synthesis technology have been developed to detect these markers with high sensitivity and selectivity (**Figure 1**). In this review, we introduce the key role of RONS and other biomarkers in the early progress of AKI, and then systematically summarize the application of emerging detection technologies in RONS (**Figure 1A**), NGAL (**Figure 1B**), GGT (**Figure 1C**), KIM-1 (**Figure 1D**) and miRNA (**Figure 1E**) for early detection of AKI. In addition, we systematically summarize the application of machine learning (**Figure 1F**) in AKI prediction algorithms and specific scenarios. Finally, we provide meaningful strategies for its further development in the clinic.

## 2. Machine learning

**Figure 2:**
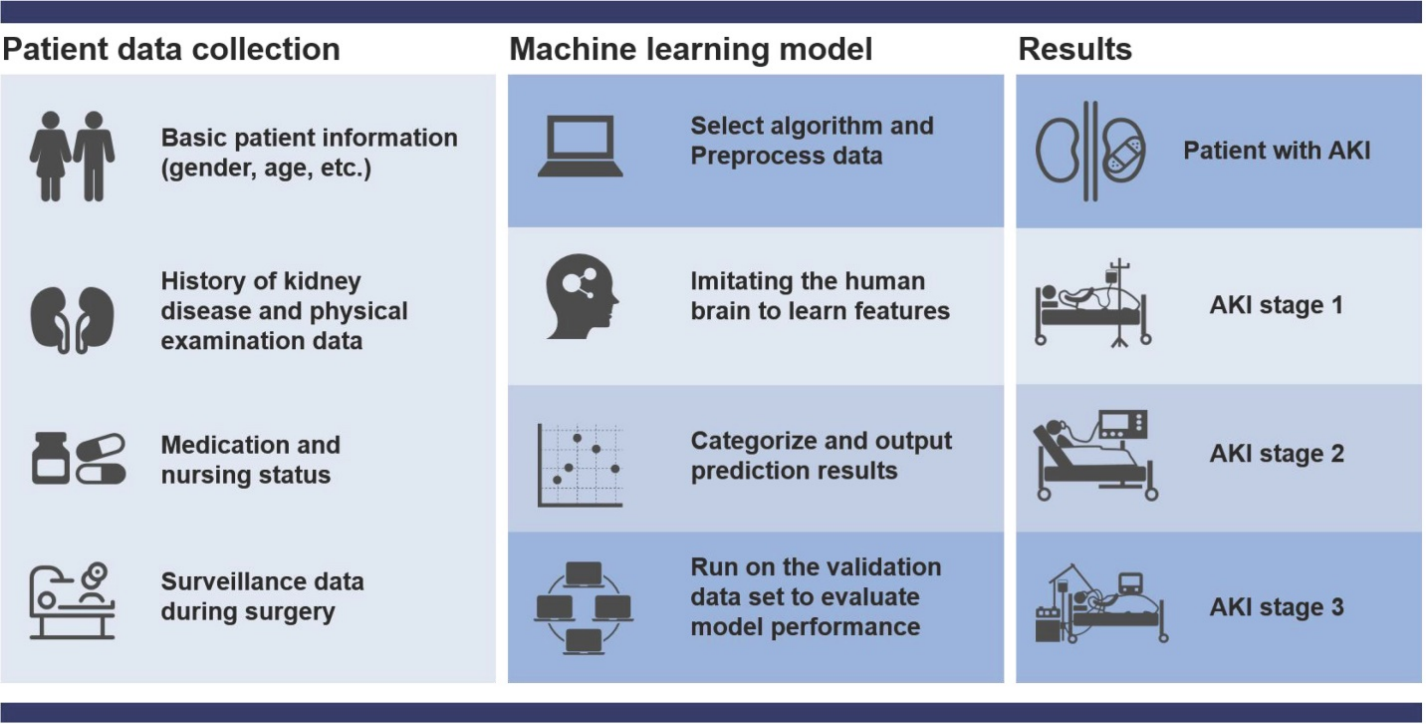
Flow chart of machine learning to predict AKI. First collect basic data, then organize the data and select the most suitable algorithm for modeling, and then continue to test and verify the model until the output is reasonable. The prediction results include the probability of patients with various grades of AKI (stage 1-3).

Currently, AKI is difficult to be diagnosed early. Even experienced clinicians cannot guarantee the accuracy of the diagnosis of AKI in patients because AKI involves a series of complex changes that vary from patient to patient. Machine learning focuses on algorithms capable of learning based on imitation of the behavior of human learning and offers promise for improving the accuracy of diagnosis of disease 31-33. Theoretically, if sufficient biomedical and patient datasets are provided, machine learning can accurately diagnose early AKI by unlocking the potential of “ground truth” data, where the correlation between data and outcomes is known (**Figure 2**). However, data collection has become a critical bottleneck for machine learning 34. On the one hand, the training effect of machine learning is limited by the size of the dataset capacity. Overfitting can occur with small datasets or simple features. When the dataset capacity is too large and has too many features, it will greatly increase the training burden and computational difficulty because there may be a linear correlation between some features. On the other hand, the way of data collection also depends on the specific situation. It is necessary to decide whether to use manual extraction of data features under the trade-off between the labeling cost of the detection results and the accuracy of the algorithm. Currently, with the widespread deployment of electronic health records (EHR), the problem of data collection has been properly solved and machine learning has had a profound impact on AKI prediction and patient monitoring. Many machine learning methods are widely developed in AKI prediction (as show as **Table 1**).

Area Under the Receiver Operating Characteristic curve (AUC) is defined as the area under the receiver operating characteristic curve (ROC). AUC is the probability that the machine learning algorithm can rank this positive sample (AKI patients) before the negative sample (non-AKI patients) 35. AUC is statistically consistent and more discriminating than other performance metrics in the evaluation of classification problems. Although we cannot arbitrarily define whether the algorithm is good or bad because the data set and processing methods are generally different, AUC provides a reasonable reference for its predictive performance. In the past 5 years, machine learning has made great progress in predicting AKI, and some models based on machine learning have very high accuracy with AUC values exceeding 0.9. According to application scope of the model, these machine learning methods are divided into preoperative AKI risk prediction, AKI prediction during surgery, postoperative AKI real-time prediction, intensive care unit AKI prediction, and AKI prediction in all hospital wards (**Table 2**).

### 2.1 Preoperative AKI Risk Prediction

Preoperative data typically include many kinds of data relate to the occurrence of AKI, such as demographic characteristics (like age, race, and sex), medical history and acuity (e.g., Charlson comorbidity index, smoking, and heart failure), physiological measurements (e.g., blood pressure, pulse, and heart rate), and type of anesthesia *etc.* Machine learning easily summarize the association between preoperative data and AKI and make accurate AKI predictions through appropriate algorithms. For example, Bihorac *et al.* developed an automated analysis framework with generalized additive model and random forest methods for the preoperative risk algorithm (MySurgeryRisk) in a single-center cohort of patients undergone major operations 47. Using the University of Florida Health Integrated Data Repository as Honest Broker, they have created a perioperative longitudinal cohort that integrated the EHR with public datasets. The number of basic features was 285, sample size was 51457 and the maximum number of feature classifications was 10,000. MySurgeryRisk calculated the risk of morbidity and mortality of 8 kinds of postoperative complications including AKI, and automatically determined the optimal threshold for dividing patients into low-risk and high-risk AKI groups and the AUC for predicting AKI was as high as 0.88. The prediction of MySurgeryRisk was very intuitive and simple. A patient whose risk score exceeded the threshold was considered a high-risk patient, and the sector representing the disease was marked in red, otherwise it was marked in green. MySurgeryRisk was also adopted as an important part of the intelligent perioperative platform, for the real-time clinical workflow of automatic surgical risk prediction to achieve prediction of AKI.

**Figure 3:**
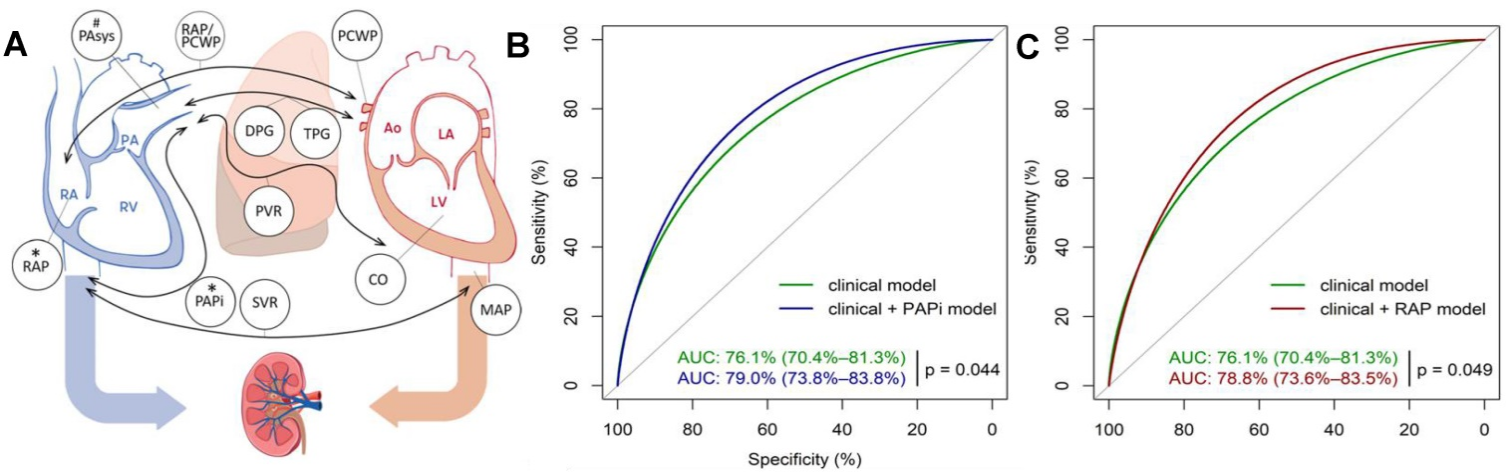
(A) Preoperative hemodynamic parameters≦30 days after heart transplantation and their relationship with postoperative right heart failure and AKI. (B) ROC curves of the clinical model (green) and clinical model + PAPI (blue) in predicting stage 3 AKI. (C) ROC curves of the clinical model (green) and clinical model + RAP (red) in predicting stage 3 AKI. Adapted with permission from 49, copyright 2018

The addition of preoperative variables with close relation to AKI further improves the accuracy of machine learning model. For example, preoperative compound hemodynamic parameters such as pulmonary artery pulsatility index (PAPI) and right atrial pressure (RAP) are closely related to AKI after heart transplantation 48. Very recently, Guven *et al.* established a model in 595 single-center cohorts with logistic regression to evaluate the effect of preoperative PAPI and RAP on AKI prediction within 30 days after heart transplantation (**Figure 3A**) 49. Patient data were obtained from the hospital database, electronic records, chart review and the catheterization reports in the Erasmus Medical Center. The results showed that the AUC of the model increased from 0.76 to 0.79 after adding the preoperative PAPI and RAP variables (**Figure 3B-C**).

### 2.2 AKI Prediction during Surgery

**Figure 4:**
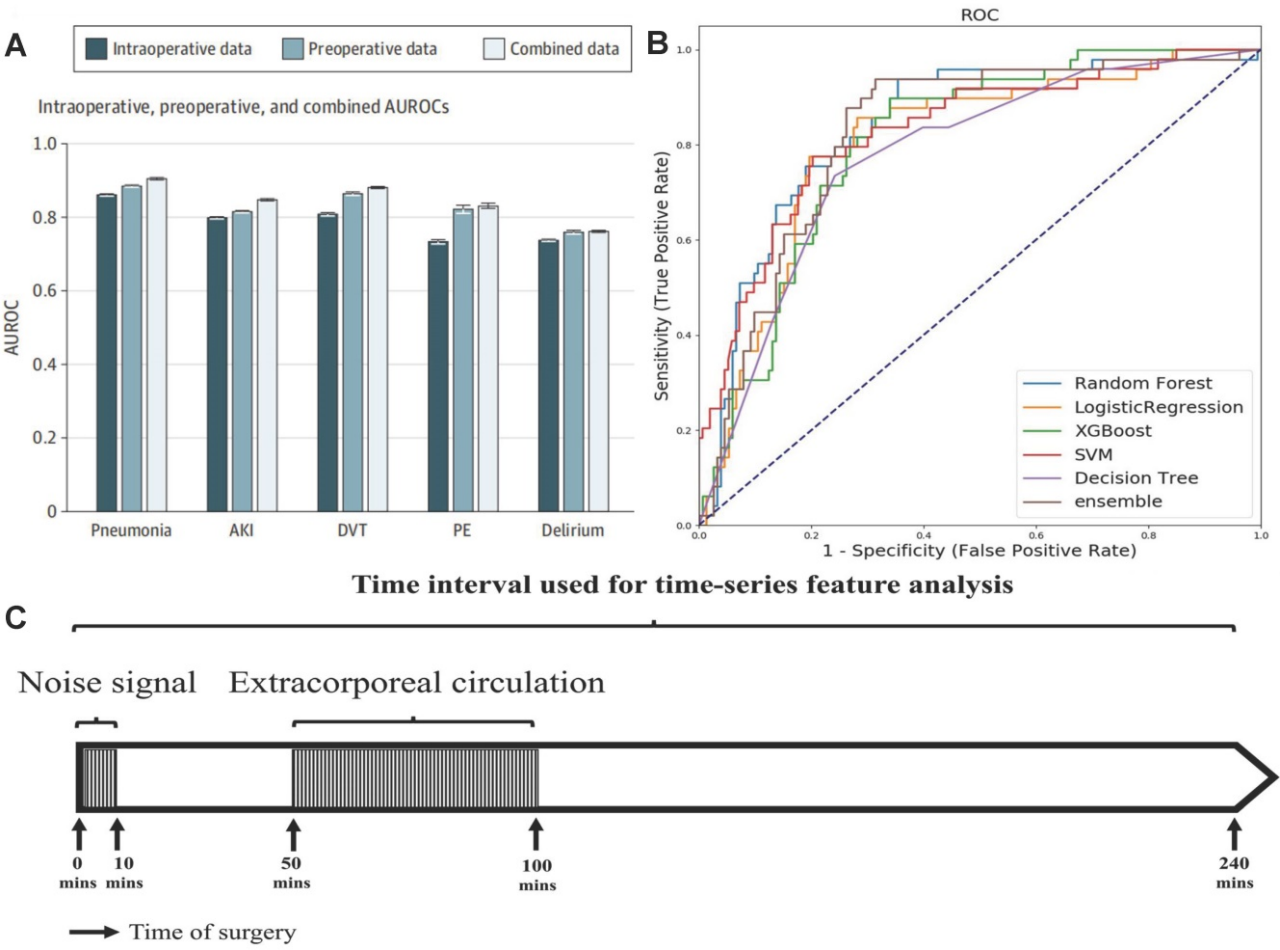
(A) AUC when using preoperative data, intraoperative data, and combined data. Adapted with permission from 50, copyright 2021 (B) Comparison of prediction performance of machine learning models. (C) Time period series feature acquisition. Adapted with permission from 51, copyright 2020

The predicting accuracy of AKI is further improved by importing the intraoperative data into the machine learning algorithm. Recently, Xue *et al.* modeled the preoperative, intraoperative, and composite data from 111888 operations performed in a single center to predict the incidence of postoperative AKI with logistic regression (LR), support vector machine (SVM), random forest (RF), gradient boosting decision tree (GBDT) and deep neural network (DNN) methods 50. The optimal hyper parameters for RF were 300 base learners, 200 maximum depth, and minimum 4 samples for splits. The optimal settings for DNN were choosing learning rate as 0.001 and batch size as 2048. Input data elements were extracted from the preoperative assessment record and anesthesia record, the target outcomes related to AKI were retrieved from EHR. For each preoperative variable, missing data were imputed using the dummy indication technique and were replaced by 0s. For each intraoperative variable, data were imputed using data-level or feature-level imputation. Among these models, the GBDT model for composite data predicted AKI most accurately, and its AUC reached 0.848. The model with only preoperative data performed better than the model with only intraoperative data, and the model with combined data performed best (**Figure 4A**). The intraoperative dataset of these models didn't include some key features, such as the description and time of the operation, blood transfusion data, urine output, and drugs. Tseng *et al.* considered the contribution of these intraoperative data to the predictive performance of model 51. They established models to predict AKI in the first week after heart surgery operating on preoperative and intraoperative data with five single methods: logistic regression, decision tree, SVM, RF, extreme gradient boosting (XGBoost) and an integrated method (RF+ XGBoost) in a single-center cohort of 671 cases. The intraoperative time series features were collected within 240 minutes after the start of the operation, excluding data between the first 10 minutes (noise signal interference) and 50-100 minutes (cardiopulmonary bypass) (**Figure 4C**), and then reduced its dimension by the principal component analysis method. Among single models, RF performed best (AUC = 0.839), decision tree performed worst (AUC = 0.781), and the predictive performance of the integrated model was better than the single model (AUC = 0.843) (**Figure 4B**). Intraoperative urine volume, intravenous infusion, blood transfusion products, and hemodynamic characteristics were found to be important factors ignored by traditional risk scoring models with shap diagram.

Similarly, Hofer *et al.* also reported a DNN model for predicting postoperative AKI with objective data available at the end of the operation in a single-center cohort of 59,981 cases 52. All data were extracted from the Perioperative Data Warehouse, which included a series of 800 distinct measures and metrics. The assessed hyperparameters were number of hidden layers (1-5), number of neurons (10-100), learning rate (0.01, 0.1), and momentum (0.5,0.9). The model evaluated the original feature set (OFS), OFS+minimum map feature (OFS+MAP) and simplified feature set (RFS) respectively. Among them, OFS+MAP had the best accuracy to predict AKI (AUC = 0.792). The multitask learning model was not superior to the single-outcome model, and the AUC value of the multitask learning model decreased even after the intraoperative hypotension duration feature was added. Even so, the predictive performance of DNN model was much better than the American Society of Anesthesiologists physical status score 53, Risk Stratification Index 54, and Risk Quantification Index 55.

### 2.3 Postoperative AKI Real-time Prediction

**Figure 5:**
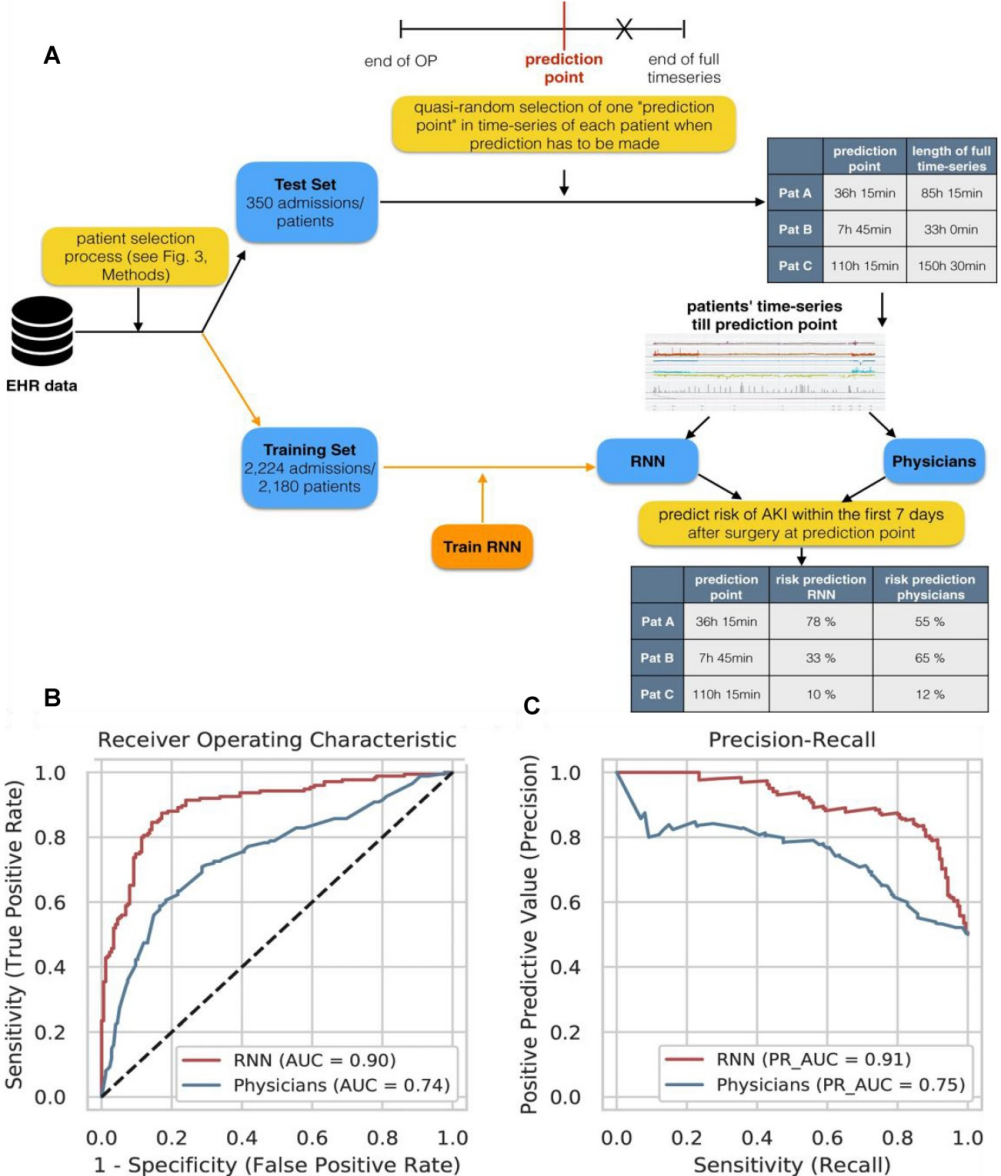
(A) Experimental design for comparing RNN model and doctor's prediction performance. (B) The ROC curve of the RNN model and the doctors. (C) RNN model and doctor's precision recall curve for predicting AKI. Adapted with permission from 56, copyright 2020

Patients are often surrounded by instrument monitoring for 24 hours after surgery, and create deluge of data all times, which provide opportunities for machine learning to monitor patient dynamics and issue AKI warnings in time by evaluating these data. For example, Rank *et al.* developed a recurrent neural network (RNN) model with 15564 single-center cohort data to predicts AKI in real time within the first 7 days after cardiothoracic surgery (**Figure 5A**), and reported the prediction results every 15 minutes 56. They retrospectively analysed EHR time series data generated at a tertiary care center for cardiovascular diseases and selected 96 routinely collected clinical parameters (static features, dynamic features, and drugs). On normal wards AKI was only defined by the creatinine criterion whereas in the recovery room or the ICU both AKI criteria (creatinine and urine) were used.

They used the Adam optimizer with a fixed learning rate of 0.001. The hyperparameter configurations with the highest overall AUC on cross-validation folds of the training set were chosen as final models. The accuracy of the RNN was much higher than that of clinicians for predicting AKI. The AUC of the model (0.851) was higher than that of the doctor (0.793) in the 2-6 hours before AKI. Moreover, the AUC of the RNN was still as high as 0.750 in the 24-168 hours before AKI, while the AUC of the clinicians was only 0.387 (**Figure 5B-C**).

### 2.4 Intensive Care Unit AKI Prediction

AKI is one of the most common acute and critical illnesses in the intensive care unit (ICU). ICU patients are under rigorous round-the-clock instrument monitoring to spawn powerful data streams for machine learning. Recently, Chiofolo *et al.* developed an AKI prediction model with the random forest method with a single center cohort to monitor the development of AKI in ICU 57. Data were abstracted from the Multidisciplinary Epidemiology and Translational Research in Intensive Care Data Mart. They used a previously validated AKI “sniffer,” an electronic tool that automatically detects AKI based on AKIN definition. The developed random forest model had 200 trees that adopted 19 different elements and sample size reached 6530. The AUC was able to reach 0.88, and AKI was detected more than 6 hours earlier than SCR in 30% of patients, and even in 53% of patients with stage 2-3. Moreover, the model provided a dynamic monitoring and nearly real-time information display for AKI in the ICU. Flechet *et al.* further evaluated the accuracy of diagnosis of AKI by the random forest analysis model and the method of detecting NGAL of AKI 58. NGAL was obtained on account of arterial blood samples which were taken upon ICU admission. Logistic regression was adopted to evaluate the predictive performance of NGAL feature compared with the admission model. The combination of NGAL and admission information significantly increased the AUC of the prediction model, and the calibration performed well. However, the decision curve showed that the improvement only occurred in the high-risk group of AKI patients. The additional cost to measure NGAL make the predictive benefit not clinically meaningful in these high-risk patients, especially in the absence of effective treatment. Similarly, Dong *et al.* also reported an interpretable AKI prediction model for pediatric ICU 59. The model was trained on an age-dependent ensemble machine learning model, which belonged to a class of models for classifications based on the sum of an ensemble of simpler 'weak classifiers'. Four types of data elements including vital signs, laboratory values, medication history, and ventilation parameters led to a total of 250 candidate predictors, and a weak classifier was learned for each predictor. The model accurately predicted moderate to severe AKI (AUC = 0.89) 48 hours prior to AKI onset with EHR data from 16863 pediatric ICU patients aged 1 month to 21 years. Remarkably, the model also provided information on the source of prediction and the intervention measures, such as “the recommended examination level and dosage for patients taking aminoglycoside drugs", which ensured clinicians to quickly intervene to reduce the risk of AKI.

### 2.5 AKI Prediction in All Hospital Wards

Recently, machine learning has also been extended to emergency departments and general wards to predict AKI. For example, Tomasev *et al.* developed a machine learning method with the U.S. Department of Veterans Affairs clinical database covering 1,239 medical institutions with more than 700,000 people 60. The clinical data were collected by the US Department of Veterans Affairs and transferred to DeepMind in a deidentified format. And they did not perform any imputation of missing numerical values. The embedding layer was of size 400 for each of the numerical and presence input features (800 in total when concatenated). The best-performing RNN architecture used a cell size of 200 units per layer and 3 layers. The AUC of the model was 0.92, and 55.8% of AKI cases were accurately predicted 48 hours in advance at the specified critical point. Each positive prediction was benchmarked against two wrong predictions, and less than 3% of inpatients were alert every day, which was suitable for low-cost but high-yield interventions. Koyner *et al.* also developed an AKI prediction model based on GBDT for all adult patients in the hospital 61. Demographics, location data, vital signs, laboratory values, interventions, medications, nurse documentation, and diagnostic orders were accessed through the Clinical Research Data Warehouse at the University of Chicago. The GBDT model identified patients with severe AKI or even RRT 1-2 days earlier than detecting SCR, and the AUC was above 0.9. In addition, the GBDT method with SCR parameters doesn't have higher accuracy for predict severe AKI than the algorithm without SCR, indicating that SCR was not always a reliable biomarker for severe AKI. Recently, Sandokji *et al.* reviewed 8473 EHRs in children younger than 18 years for pediatric early diagnosis of AKI, and they adopted a penalty level for selection of only ten variables to create a logistic regression model 62. The model predicted the risk of AKI in pediatric patients 48 hours in advance and risked stratify the results with high AUC (0.76-0.81).

**Figure 6:**
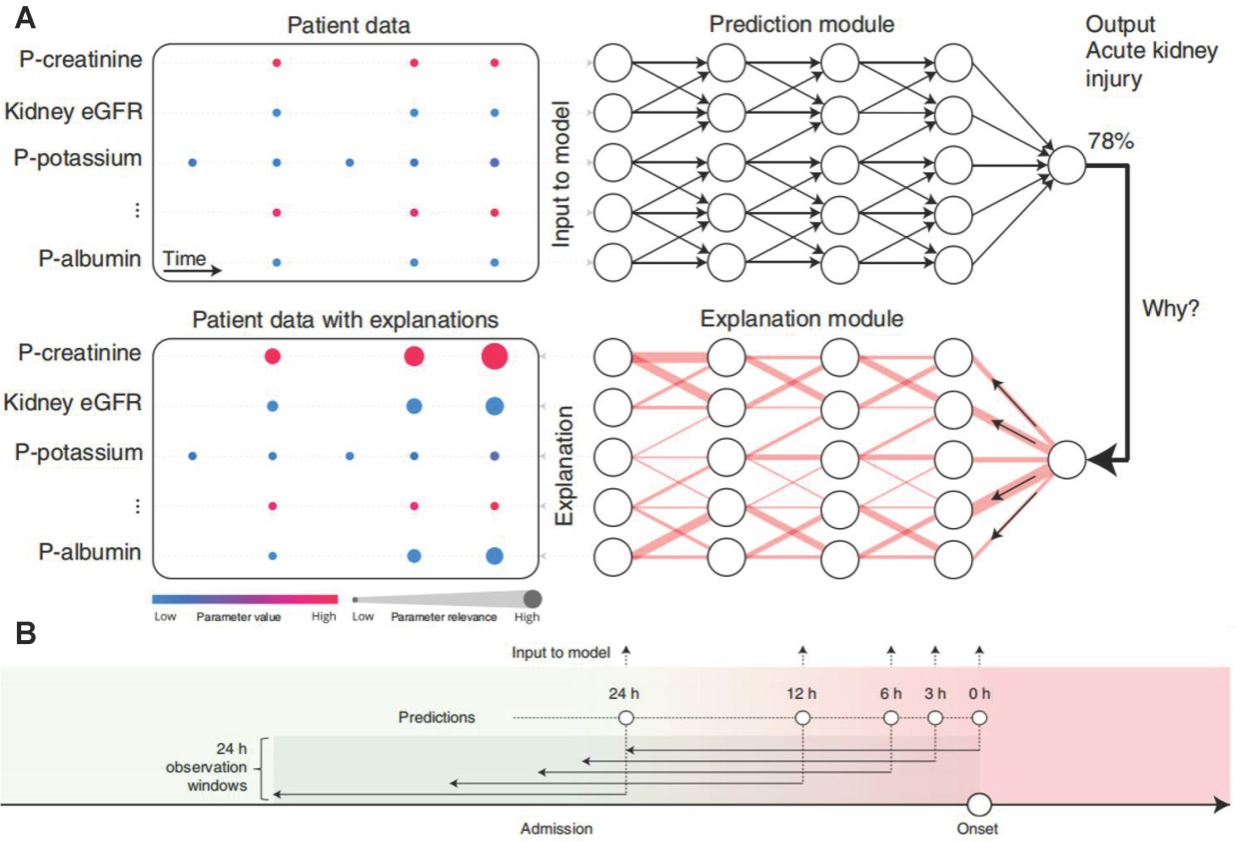
(A) Overview of XAI-EWS system. (B) The model was trained and evaluated at 0, 3, 6, 12 and 24 hours before the onset of AKI. Each model has a 24-hour retrospective observation window. The color gradation from green to red indicates continued deterioration to AKI. Adapted with permission from 66, copyright 2020

Most of these models are black-box predictions that cannot readily be explained to clinicians. Transparency and interpretability are necessary for the widespread introduction of artificial intelligence models into clinical practice 63-65. The above-mentioned weak classifier ranking and shape interpretation methods not consider the dependence between variables, which inevitably lead to the correlation bias. In order to better explain the AKI prediction machine learning model, Lauritsen *et al.* developed the AKI Early Warning Score (XAI-EWS) to provide a simple visual explanation for the predictions 66. XAI-EWS consisted of a temporal convolutional network (TCN) prediction model and a deep Taylor decomposition (DTD) interpretation module (**Figure 6A**), in which TCN operated sequentially on individual EHRs with in the predictions range of 0-100%, and the DTD explanation module delineated the TCN predictions in terms of input variables by a decomposition of the TCN output on the input variables. The model was trained to optimize the crossentropy loss using the Adam optimizer with mini-batches of the size of 200, a learning rate of 0.001, and a dropout rate of 10%. The AUC and precision recall curve (PRC) of XAI-EWS performed well compared with sequential organ failure assessment scores, modified early warning score systems, and gradient boosting vital sign model models. The AUC of XAI-EWS ranged from 0.79 to 0.88 during the 24 hours before the onset (**Figure 6B**) and XAI-EWS also were explained to the clinicians which relevant EHR data the prediction results were based on from a global perspective and a single patient perspective, respectively.

Transportability is a very important aspect of model application. Recently, Song *et al.* developed an interpretable GBDT model with 153821 cases of EHR data from a source healthcare system to calculate AKI risk over the next 48 hours from admission to discharge for all hospitalized patients 67. They tuned the hyperparameters (depth of trees: 2-10; learning rate: 0.01-0.1; minimal child weight: 1-10; the number of trees was determined by early stopping, i.e., if the holdout area-under-receiver-operating-curve had not been improved for 100 rounds, then we stopped adding trees) within training set using 10-fold cross validations. The GBDT model was further externally verified by the Shap value (the difference in feature selection between different sites) from five other medical systems, and the source system model had reduced predictive performance in a new medical system, possibly due to demographic factors as well as differences in data description. A statistical tool adjMMD was developed to find the source of data heterogeneity for improving transportability. Other medical systems adjusted the input characteristics according to the statistical results of adjMMD, and more accurate prediction results were obtained by the source system AKI prediction model.

## 3. RONS Imaging Probes

**Figure 7:**
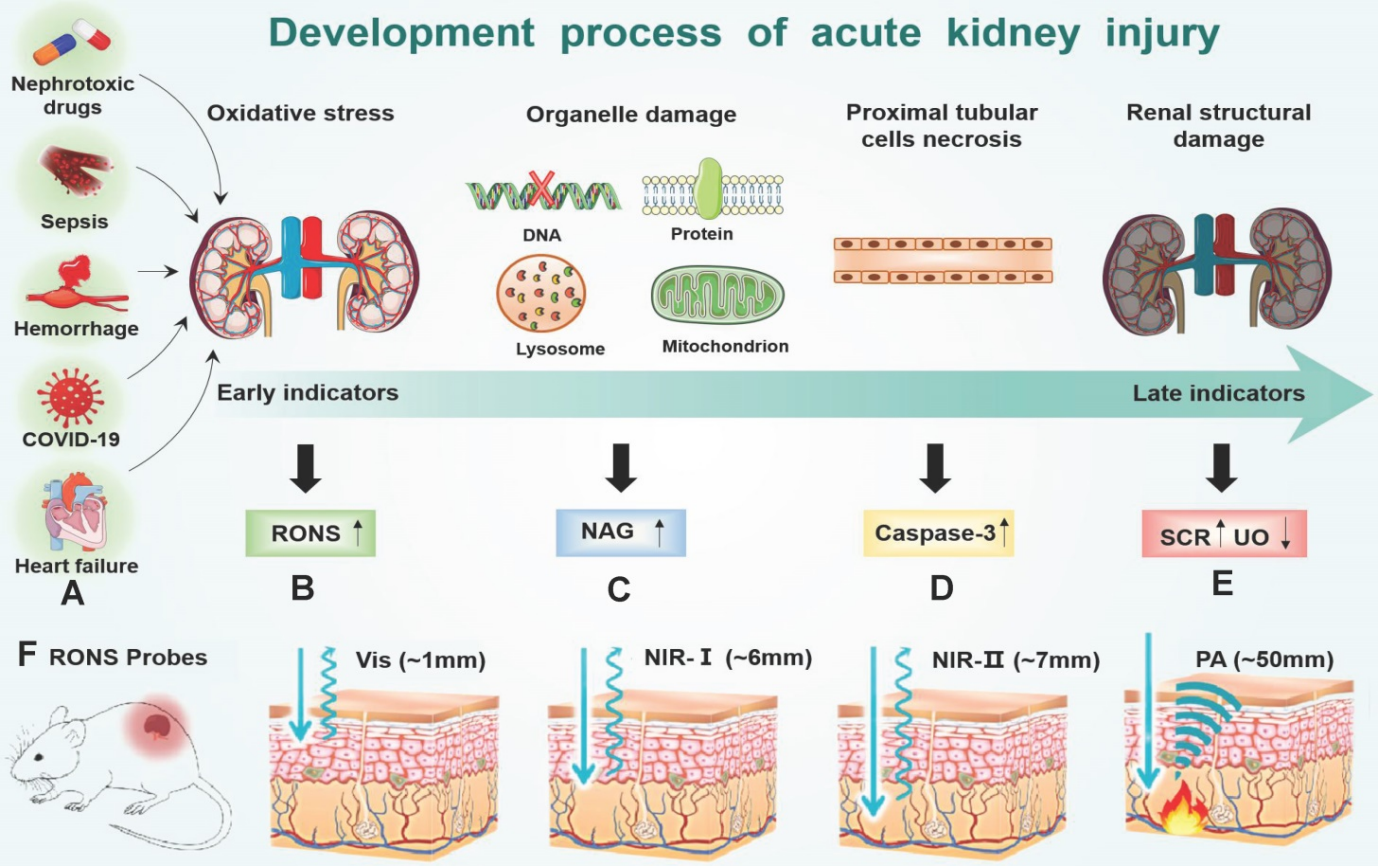
** (**A) Many factors (hemorrhage, heart failure, nephrotoxic drugs, sepsis and COVID-19) accumulate RONS burst in the early of AKI stage (B). RONS damages the lysosomal membranes of the proximal tubular cells, leading to an increase in the concentration of (C) NAG in the kidney. After the organelles are destroyed by RONS, the proximal tubular cells are necrotic, which leads to an increase in the concentration of (D) caspase-3 in the kidney and ultimately leads to the destruction of the metabolic function of the kidney, and changes of (E) SCR and UO. (F) Imaging depth of different RONS probes: visible light (~1mm) NIR-Ⅰ (~6mm) NIR-Ⅱ (~7mm) PA (~50mm).

Oxidative stress plays a major role in the early progression of AKI caused by various factors (**Figure 7A**). Here, we take drug-induced AKI as a typical example because drug-induced AKI is the most common clinically (accounting for about 20%). Moreover, the drug-induced AKI animal model is widely used at present, which is highly like human AKI lesions and is closer to the pathophysiological process of AKI. For example, cisplatin is very potent chemotherapeutics with strong nephrotoxicity, which are actively transported to the proximal tubular cells through the organic cation transporter 2 in cisplatin-induced AKI^68^. Cisplatin is hydrolyzed into positively charged electrophile molecules to destroy the mitochondrial respiratory chain complex to increase O_2_^.-^ production in the proximal tubular cells 69-72. In addition, cisplatin inhibits mitochondrial transcription factors through the up-regulation of miRNA-709, which also lead to the decline of mitochondrial function and ultimately increases the production of O_2_^.-^ 73, 74. O_2_^.-^ can further generate other RONS, such as H_2_O_2_, ^.^OH, ONOO^-^ and hypochlorous acid (HOCl) 75, 76. Furthermore, cisplatin decrease expression of endogenous antioxidant enzymes (e g. superoxide dismutase, and catalase) to increases intracellular accumulation of RONS (**Figure 7B**). Finally, excessive RONS lead to organelle membrane damage, DNA strand break and protein denaturation through direct oxidation of lipids, nucleic acids, and proteins 77-81. In particular, RONS lead to an increase in the concentration of N-acetylglucosamine (NAG) and caspase-3 in turn by damaging the lysosomal membranes of the proximal tubular cells in the kidney 82 (**Figure 7C-D**). Last, many cell apoptosis in the kidney eventually leads to the destruction of the metabolic function of the kidney, and changes of SCR and UO 83 (**Figure 7E**). According to the above analysis, the sequence of biomarker changes in the early AKI process is RONS, NAG, and caspase-3. SCR and UO only change when renal organic performance changed, which is too late to lose the value of early diagnosis. Therefore, the accurate detection of renal RONS can achieve the early diagnosis of AKI, which is essential for promptly initiating renal protective interventions to prevent the transition to more serious complications.

Traditional methods (like fluorescent probes, chemical analysis, electron spin resonance, *etc.*) 84-88 are difficult to detect RONS in the kidney because of their significant limitations such as poor tissue permeability and low kidney-specific distribution. The detection of RONS in the kidney must meet the following requirements. First, the detection signal of the probes can penetrate the tissue to achieve accurate quantitative measurement because the kidney is located deep inside the body (**Figure 7F**). Second, the probes must be effectively taken up by the kidney to avoid signal interference from other organs 89. In recent years, many probes have been developed to detect RONS by overcoming the shortcomings of traditional methods. These methods are mainly divided into near-infrared fluorescence (NIF) imaging, chemiluminescence imaging and photoacoustic (PA) imaging (**Table 3**). Compared with traditional methods, these methods can achieve timely diagnosis, high imaging depth, high renal clearance, good biological safety, low background noise, and real-time detection.

### 3.1 NIF Imaging

Conventional fluorescence imaging generally adopts visible light as the excitation light source, and the generated fluorescence is usually located in the visible light region 90. The high absorption and scattering of these lights by human tissues lead to a short penetration depth of conventional fluorescence imaging 91. NIF imaging can overcome the penetrability limitations of deep tissue imaging because the near-infrared light (650-2000nm) have much lower absorption and scattering than visible light in the human tissues(**Figure 7F**) 92. In addition, the NIR probe must be highly hydrophilic to prevent it from being captured by the reticuloendothelial system to increase the specificity of the kidney. These highly specific NIF probes have been adopted for imaging of O_2_^.-^, H_2_O_2_, and ONOO^-^ in the AKI site. For example, Pu *et al.* developed a series of near-infrared optical imaging probes (MRPs1-3) based the cyanine fluorescent dye (CyOH) for imaging of O_2_^.-^, NAG and caspase-3 in kidney respectively 93. MRPs1-3 consisted of three parts, namely a renal clearance functional group ((2-hydroxypropyl)-β-cyclodextrin, HPβCD), a fluorescent substrate CyOH and a specific recognition group. A good kidney probe usually needed a high renal clearance rate that was, probe molecules avoided entering other organs, enriched in the kidney, and finally was excreted through the urinary system. HPβCD had a suitable molecular weight for glomerular filtration and high hydrophilicity for promoting renal clearance efficiency. The 24h renal clearance rate of HPβCD-substituted CyOH was 97±2.7%, which was much higher than that of methyl-substituted CyOH (about 5%). In order to realize the specific imaging of O_2_^.-^, NAG and caspase-3 in kidney, the hydroxyl group on the benzene ring of CyOH was connected with diphenyl phosphine, N-acetyl-β-d-glucosamine and tetrapeptide sequence (Asp-Glu-Val-Asp) respectively. After these specific groups on the phenolic hydroxyl group was specifically cleaved by O_2_^.-^, NAG, and caspase-3, the fluorescence in the near-infrared region (720nm) increased about 20 times. MRPs 1-3 were effectively enriched in the kidney to achieve the imaging of O_2_^.-^, NAG and caspase-3. Imaging of O_2_^.-^ by MRP1 could be very effective in diagnosing early AKI. The O_2_^.-^-based imaging had the best results, and identified AKI 4, 36 and 60 hours earlier than NAG, caspase-3 and clinical methods(SCR/UO), respectively.

In addition to O_2_^.-^, other RONS are also used for early diagnosis of AKI through NIF imaging, like H_2_O_2_ and ONOO^-^. Recently, Xu *et al.* developed a NIF nanoprobe (TA-TPABQ) to detect H_2_O_2_ in ischemic AKI 94. TA-TPABQ was composed of a hydrophobic photosensitizer (1-(3-boronbenzyl)-4-(2-(4'-(diphenylamine)-[1,1'-biphenyl]-4-Base) vinyl) quinoline-1-salt, TPABQ) and hydrophilic natural polyphenol tannic acid (TA). H_2_O_2_ cleaved the borate bond formed by the reaction of TPABQ and TA specifically, then activated the probe to obtain the activated product (4-(2-(4'-(diphenylamine)-[1,1'-biphenyl]-4-yl) vinyl)-1-(3-hydroxybenzyl) quinoline-1-salt, TPAQ-OH). TPAQ-OH had the feature of aggregation-induced emission, and could emit NIF at 725nm. After TA-TPABQ was injected intravenously into ischemic AKI mice, the fluorescence signal of ischemic kidney had a significant increase compared with a healthy kidney. In addition, cell activity was not affected even when the nanoprobe's concentration reached 60 μM. Recently, Wang *et al.* designed a kidney-targeting NIF probe (KNP-1) to detect ONOO^-^ for early diagnosis of AKI 95. KNP-1 had two key building blocks, a Nile red derivative fluorophore and an ONOO^-^ recognition group. The Nile red derivative was selected as the NIF because it was quickly distributed in the kidney, but almost no signal in other tissues. KNP-1 was rapidly distributed in the kidney, and its renal clearance rate exceeded 90% at 3 hours after injection, and was almost 100% after 24 hours. High concentration ONOO^-^ restored the fluorescence (679nm) of Nile red derivatives by oxidizing p-hydroxy aniline in AKI.

Very recently, near-infrared zone II (1000-1700nm, NIR Ⅱ) probes and multiphoton probes with deeper penetrating ability have also been developed for RONS imaging of AKI sites. Fluorescent probes in the near-infrared zone II (1000-1700nm, NIR Ⅱ) have lower background interference than the probes in the near-infrared zone I (750-900 nm, NIR I) (**Figure 7F**) 96-98. Chen *et al.* developed a kidney targeting peptide coupled NIR Ⅱ probe (KTP5-ICG-GNP) to diagnose AKI early with low spontaneous fluorescence background 99. The KTP5-ICG-GNP probe was composed of three parts: the kidney targeting polar peptide (KTP5), the NIR Ⅱ fluorescent signal molecule indocyanine green (ICG) and the fluorescence quencher gold nanoparticles (GNP). RONS oxidized the Au-S bond between GNP and ICG-KTP5 to restore ICG fluorescence at site of AKI. The KTP5-ICG-GNP probe detected the AKI 48 hours earlier than SCR in the cisplatin-induced AKI mouse model. Multiphoton fluorescence is anti-stocks shift fluorescence method, which emits short-wavelength visible light by near-infrared light as the excitation light source. Therefore, multiphoton fluorescence has deeper tissue penetrating ability than traditional fluorescence imaging 100, 101. Lv *et al.* developed a mitochondrial-targeted two-photon fluorescent probe Naph-O_2_^.-^ to diagnose AKI by detecting renal O_2_^.-^. The Naph-O_2_^.-^ probe was composed of three parts, h-hydroxynaphthalimide as the two-photon fluorophore, trifluoromethanesulfanote as the O_2_^.-^ response group, and triphenylphosphous as the mitochondrial targeting group. Naph-O_2_^.-^ were activated by O_2_^.-^ to emit strong fluorescence (500-550nm) under the excitation of 800nm illuminant, and the detection limit was as low as 0.39μmol. Naph-O_2_^.-^ detected AKI induced by cisplatin in mice 48 hours earlier than SCR, with an imaging depth of 130μm. In addition, the probe exhibited low cytotoxicity to live HepG2 cells when evaluated by MTT assays.

### 3.2 Chemiluminescence Imaging

Different from NIF imaging, chemiluminescence imaging directly emit light through chemical reactions with little tissue scattering and no interference from self-luminescence. Therefore, chemiluminescence imaging is more sensitive and deeper tissue penetration depth than NIF 102-105. For example, Pu *et al.* developed a dual-channel molecular optical imaging probe (MRPD) with chemiluminescence and NIF groups for O_2_^.-^ imaging in early AKI^93^. MRPD contained trifluoromethanesulfonate substituted phenoxy dioxane as a chemiluminescent signal. O_2_^.-^ specifically cleaved the trifluoromethanesulfonic acid of MRPD to form a phenate dioxane unstable intermediate, which spontaneously emitted 540nm light. In addition, MRPD contained a non-shell heptamethine cyanine dye as a fluorophore with a NIF at 760nm, which enabled MRPD to detect changes in glomerular filtration rate (GFR). The dual-channel molecular optical imaging endowed MRPD to analyze the correlation between renal O_2_^.-^ and GFR changes. MRPD detected the occurrence of AKI before the GFR decreased. In DTZ and cisplatin-induced AKI, MRPD detected AKI 16 hours and 60 hours earlier than the SCR method, respectively. Recently, Huang *et al.* further developed a near-infrared-based chemiluminescence imaging (NCR1-2) for the diagnosis of early AKI 106. NCR1-2 contained two functional groups, namely HPβCD to improve renal clearance efficiency and dicyanomethylene-4-hydro-pyran modified with sap dioxane as the near-infrared chemiluminescence moiety. NCR1was linked to trifluoromethanesulfonate ester that specifically cleaved by O_2_^.-^, and NCR2 was linked to formate that specifically cleaved by ONOO^-^. After cleavage, the remaining unstable intermediate emitted chemiluminescence at 700nm. After tail vein injection of NCR1-2 into mice, NCRs were effectively enriched in the kidney to achieve the imaging of O_2_^.-^ and ONOO^-^. The O_2_^.-^ based NCR1 was very sensitive in the early detection of AKI, and detected cisplatin-induced AKI 16 hours and 60 hours earlier than the ONOO^-^ based NCR1 and immunofluorescence and H&E staining, respectively.

### 3.3 PA Imaging

**Figure 8:**
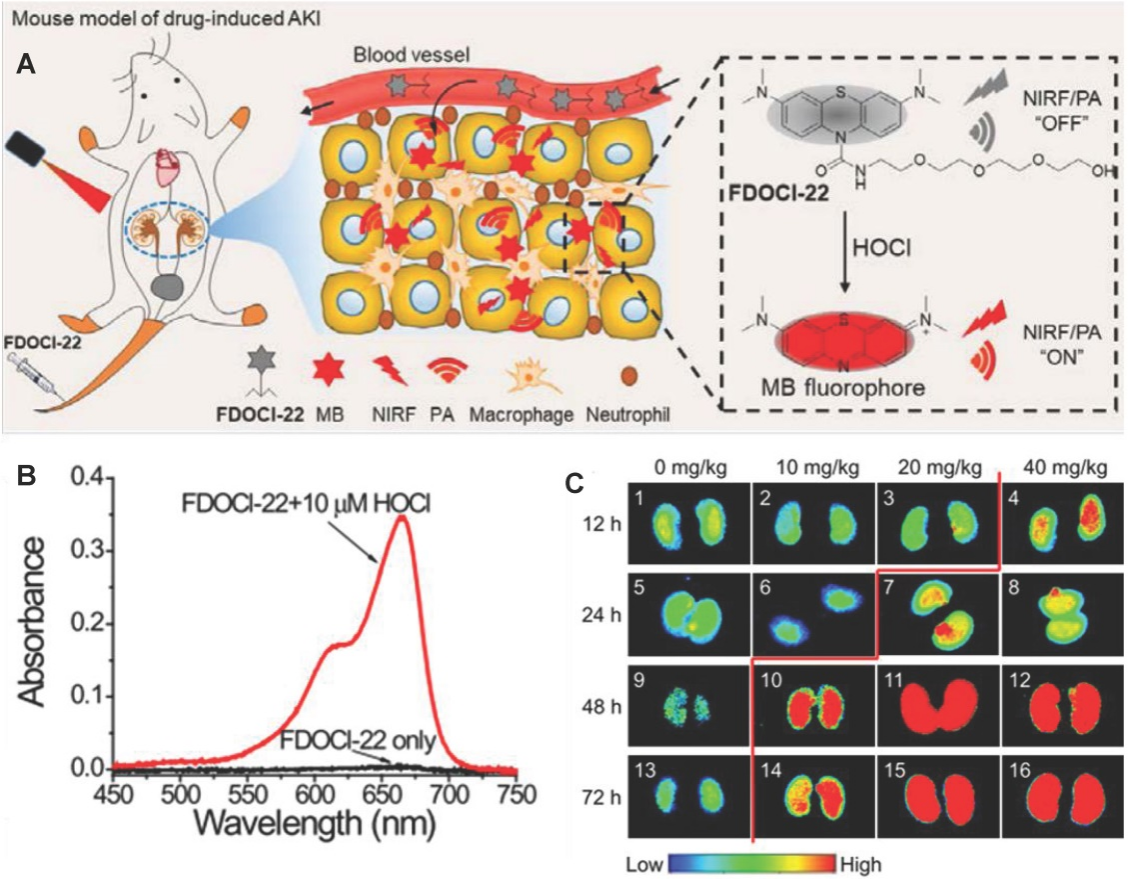
(A) Structure of FDOCl-22 and its detection mechanism. (B) Absorption spectra of FDOCl-22 before and after adding HOCl (10 μM). (C) Fluorescent images of the kidney of a series of mice intraperitoneally injected with cisplatin of varying concentrations for different time periods and then intravenously injected with FDOCl-22 (200 μL × 0.5 mM) and average fluorescence intensity output of the groups (2.5 μM) to ONOO^-^(0-20 μM). Adapted with permission from 108, copyright 2020.

PA imaging as a mixed-mode imaging method based on the photoacoustic effect, has better spatial resolution and higher penetration depth than optical imaging (**Figure 7F**) 97, 107. Recently, Tao *et al.* developed a PA and NIF molecular probe (FDOCl-22) to detect HOCl in drug-induced AKI 108 (**Figure 8A**). FDOCl-22 had two special functional modules: methylene blue as a near-infrared light absorber and near-infrared fluorophore, and a short chain of hydrophilic polyethylene glycol as the renal clearance part 109. HOCl specifically cleaved the amide bond in FDOCl-22 and caused the methyl bromide fluorophore to be released, which restored the light (at 680nm) absorption and the NIF (640-800nm) of methyl bromide fluorophore (**Figure 8B-C**). Based on the principle, the FDOCl-22 could perform dual-modal imaging of NIF and PA for early AKI by detecting HOCL. Specially, the PA signal (excitation illuminant at 680nm) in the kidney was detected 10 minutes after FDOCl-22 injection, and the PA signal intensity gradually increased within 40 minutes. Moreover, FDOCl-22 detected cisplatin-induced AKI at least 24 hours earlier than the SCR-based method. Biocompatibility studies indicated that FDOCl-22 had no toxic effects on RAW264.7 cells and did not induce any obvious pathological damage to major organs.

Zhang *et al.* further developed a SiRho-HD probe that combined ratiometric NIF and PA to ONOO^-^ imaging for the early diagnosis of AKI 110. Ratiometric NIF self-calibrated through the two emission peaks of NIF to obtain more reliable results. SiRho-HD was prepared by connecting homodimer dyes (HD) and Si-rhodamine with a short piperazine-based flexible carbon chain. Si-rhodamine had good fluorescence brightness and a high renal clearance rate due to its excellent water solubility and small molecular weight. ONOO^-^ specifically cleaved HD dye to break Förster resonance energy transfer (FRET) between HD dye and Si-rhodamine, and then the ratio between Si-rhodamine fluorescence (at 680nm) and HD dye fluorescence (at 750nm) changed. Moreover, the light absorption (at 719nm) of HD disappeared, and the PA signal (exciting with 715nm light) of SiRho-HD also decreased 111. A MTS assay result suggested good biocompatibility of SiRho-HD. After intravenous injection of SiRho-HD into cisplatin-induced AKI mice, the ratio of 680nm/750nm NIF intensity was higher than in healthy mice. More importantly, the PA signal was reduced by 1.95 times in AKI mice, while healthy mice did not change. The significant reduction of PA signal and change of the NIF intensity ratio ensured very accurate detection of early AKI.

## 4. NGAL

NGAL is expressed at low levels in renal tubular epithelial cells under normal physiological conditions. The level of NGAL in blood increases significantly within 2-6 hours after AKI 112, 113. In a prospective study, Chui *et al.* demonstrated that the AUC of NGAL was ≥ 0.73 to detect AKI at 3 days before AKI onset 114. Jahaj* et al.* also proved NGAL was more accurate for predicting AKI development than creatinine^115^. Currently, NGAL is still measured by the classical ELISA method. However, ELISA have many shortcomings for NGAL detection, such as complicated sample preparation and detection process, long analysis time, high cost and unstable antibody, and false positive results 116-118. In recent years, innovative materials and new detection principles have been developed to improve the stability, and convenience of NGAL detection. These emerging NGAL detection methods are mainly divided into three categories: electrochemical immunosensor, SPR biosensor and Raman spectroscopy specific immunoassay.

### 4.1 Electrochemical Immunosensor

**Figure 9:**
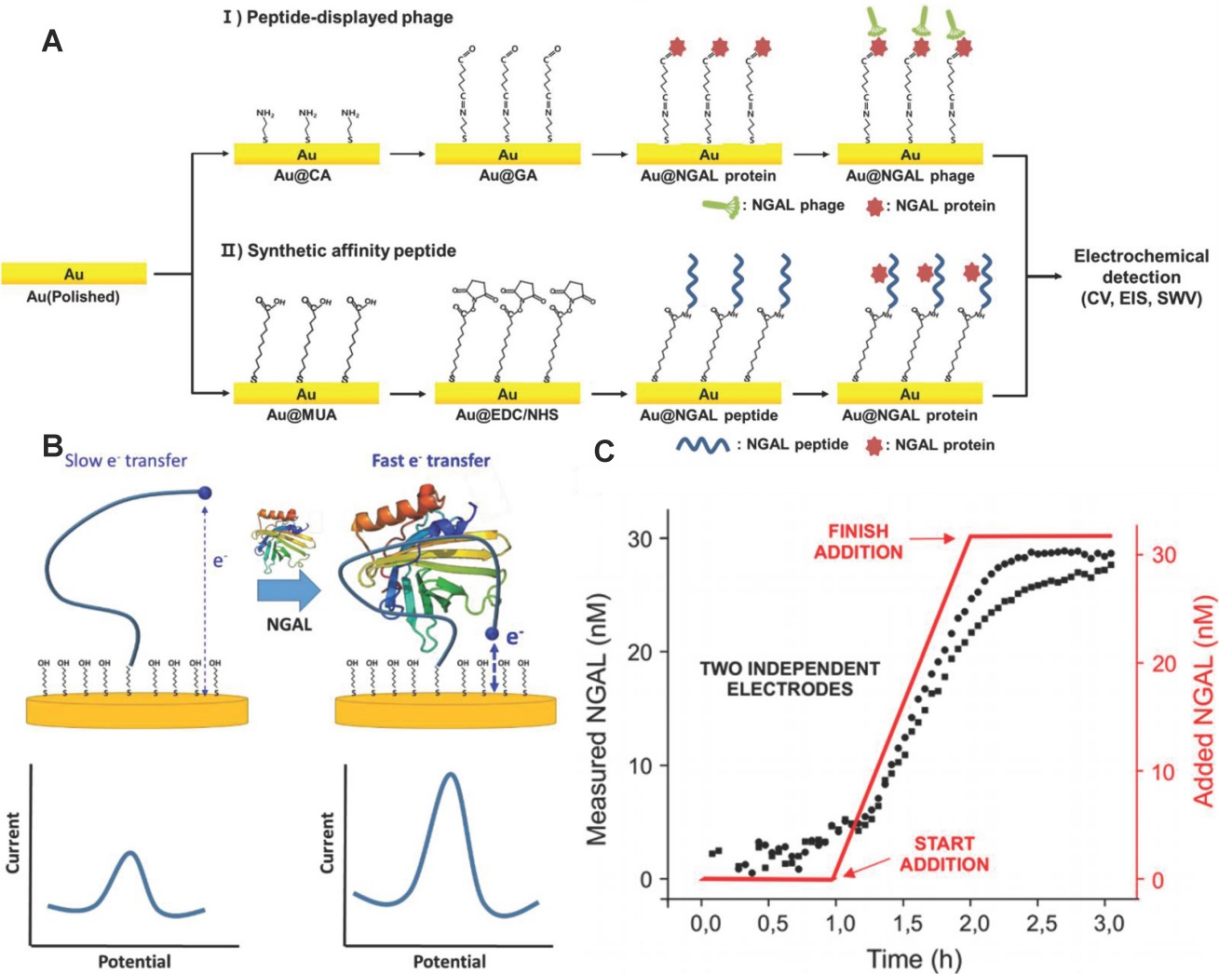
(A) A schematic illustration of the electrochemical sensor showing the principles of peptide sensors. Adapted with permission from 127, copyright 2019 (B) The EAB sensor uses aptamers combined with NGAL to induce folding to generate easily measurable, fast and reversible electrochemical signals, without the need for external reagents or washing steps, so as to achieve continuous and real-time molecular monitoring. (C) Monitor the NGAL concentration within 3 hours with a resolution of 3 minutes. Adapted with permission from 129, copyright 2020

Electrochemical immunosensor have the advantages of simplicity and short detection time, and was especially suitable for AKI detection 119-122. Traditional electrochemical immunosensors adopt enzyme-labeled NGAL antibodies and electrodes modified with nanomaterials to improve the sensitivity and selectivity of NGAL detection 123-126. However, the NGAL antibodies are susceptible to inactivation due to changes in environmental temperature, pH and other conditions. Recently, Cho *et al.* developed an NGAL electrochemical immunosensor with the specific affinity peptide NGAL BP1 (amino acid sequence is DRWVARDPASIF) instead of NGAL antibodies as biomolecular recognition elements 127 (**Figure 9A**). NGAL BP1 exhibited stronger stability than NGAL antibodies, and further introduced cysteine at the C-terminus of the peptide to form a thiol self-assembled monomolecular membrane at the surface of the gold electrode. The combination of NGAL and NGAL BP1 significantly increased the impedance of the gold electrode. The quantification of NGAL was achieved according to the linear change of electrochemical impedance and NGAL concentration. The detection limit of the sensor was 1.74 ng/mL, which was comparable to commercial ELISA detection kits. Importantly, the method took only about 2 h to analyze and was significantly shorter than ELISA.

Nucleic acid aptamer also has higher stability compared with NGAL antibody, and specifically bind to NGAL through its own reversible conformational change 128. Recently, Parolo *et al.* developed an electrochemical sensor EAB based on the NGAL aptamer placed on the patient 129. The EAB sensor not only was reused many times, but also continuously measured NGAL for 12 hours. The 5'end and 3' end of the aptamer were modified with methylene blue (as a signal transducer) and thiol (as a linker to the gold electrode), respectively. The conformational change of the NGAL aptamer caused by the combination of NGAL and the aptamer promoted methylene blue closer to the gold electrode, and generate electrochemical signals by increasing the electron transfer rate (**Figure 9B**). The response time of the EAB sensor was only about 1 minute. The EAB sensor monitored the NGAL in real urine samples with a time resolution of sub-minute (**Figure 9C**).

### 4.2 SPR

**Figure 10:**
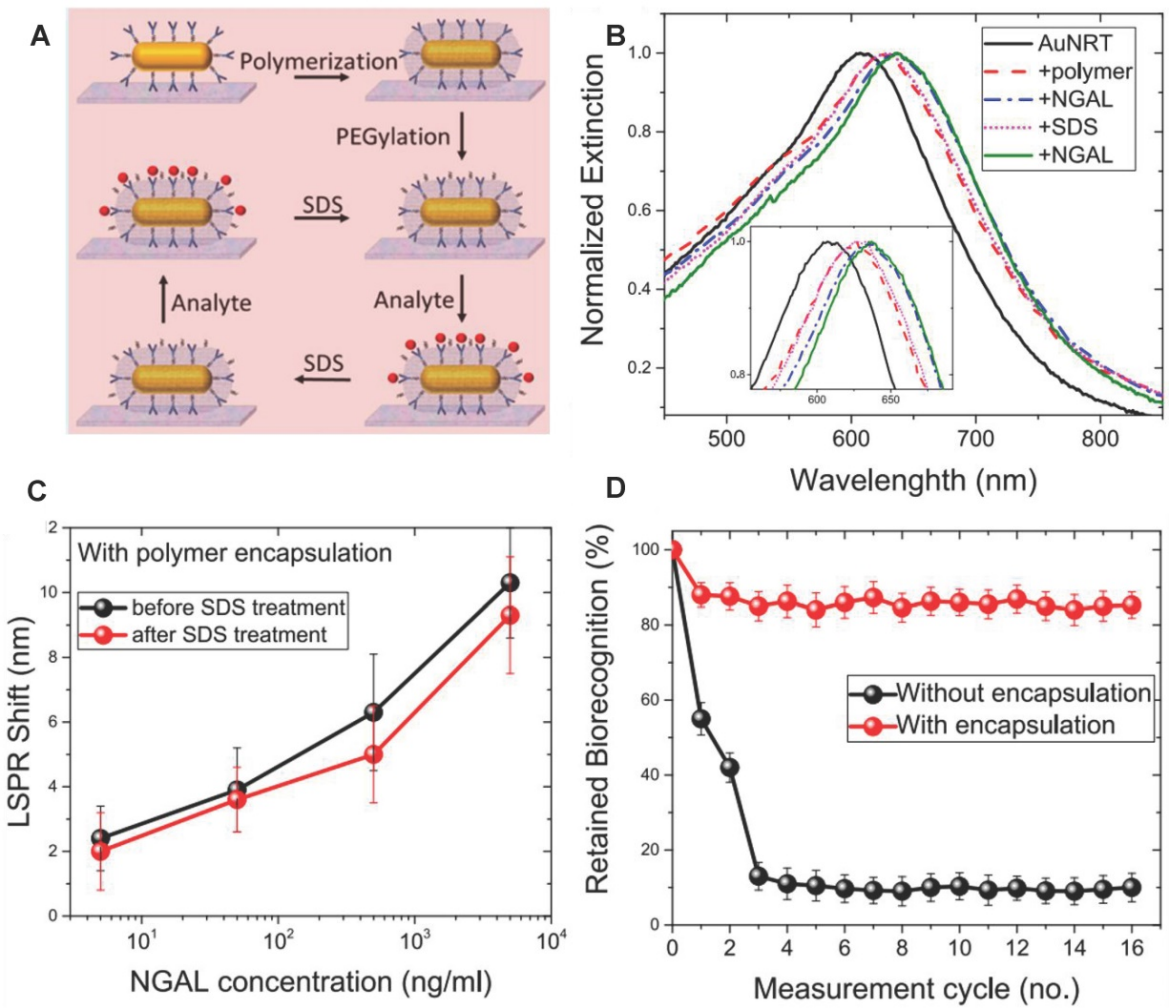
(A) Schematic illustration of the steps involved in the organosilica-based biopreservation of bioconjugates to realize refreshable biosensors. (B) Extinction spectra corresponding to each step involved in the polymer encapsulation strategy of AuNRT-NGAL antibody bioconjugates. The inset shows zoomed-in spectra highlighting the shifts in the LSPR wavelength. (C) LSPR shift upon exposure of polymer-encapsulated AuNRT-NGAL antibody bioconjugates to different concentrations of NGAL before and after SDS treatment. (D) Retained biorecognition capability of biosensors with and without polymer encapsulation over multiple capture/release cycles of NGAL. Adapted with permission from 133, copyright 2020

SPR method has the advantages of real-time, fast, and high sensitivity for NGAL detection compared with the traditional ELSA method 130-132. Recently, Gupta *et al.* developed reusable SPR biosensor based on the NGAL antibody 133 (**Figure 10A**). NGAL antibody was modified on gold nanorods, and then adopted silica shell to maintain the stability of the NGAL antibody by copolymerization of (3-aminopropyl)-trimethoxysilane and trimethoxy (propyl) silane around the NGAL antibody. The local SPR (LSPR) wavelength of Au NRs red-shifted because of the increasing refractive index of the surrounding medium when NGAL was captured by the NGAL antibody (**Figure 10B**). The silicone-coated antibodies still maintained nearly 80% biorecognition ability after undergoing 16 capture / release cycles, in sharp contrast to the uncoated antibodies that maintained less than 20% recognition ability after only 3 capture / release cycles (**Figure 10C-D**). The SPR biosensor had a very high sensitivity, and its minimum detection limit was 40 ng/mL.

### 4.3 Surface Enhanced Raman Spectroscopy (SERS)

NGAL has three molecular forms: monomer (~25kDa), disulfide bond-linked homodimer (~45kDa) and heterodimer NGAL/MMP-9 (~135kDa). Among them, only the NGAL monomer is the specific biomarker of AKI 134. However, most of the current methods can't effectively distinguish these molecular NGAL forms and easily lead to false positives in the AKI diagnosis. Recently, Jiang *et al.* established a NGAL molecular form-specific detection method by SERS 135. The SERS method adopted the NGAL antibody modified 4-mercaptobenzoic acid (MBA)-Ag nano-monolayer film as the SERS enhancing substrate. After the monomeric NGAL was captured by the SERS enhancing substrate, typical Raman peak of MBA (1075 cm^-1^) was red-shifted by relaxing MBA to a certain extent, while the homodimer NGAL caused the blue shift of the MBA Raman peak by stretching of MBA. The SERS method effectively distinguished the molecular form of NGAL and reduced the false positive detection of AKI. The detection limit of the two forms of NGAL was as low as 10 ng/mL.

## 5. GGT

**Figure 11:**
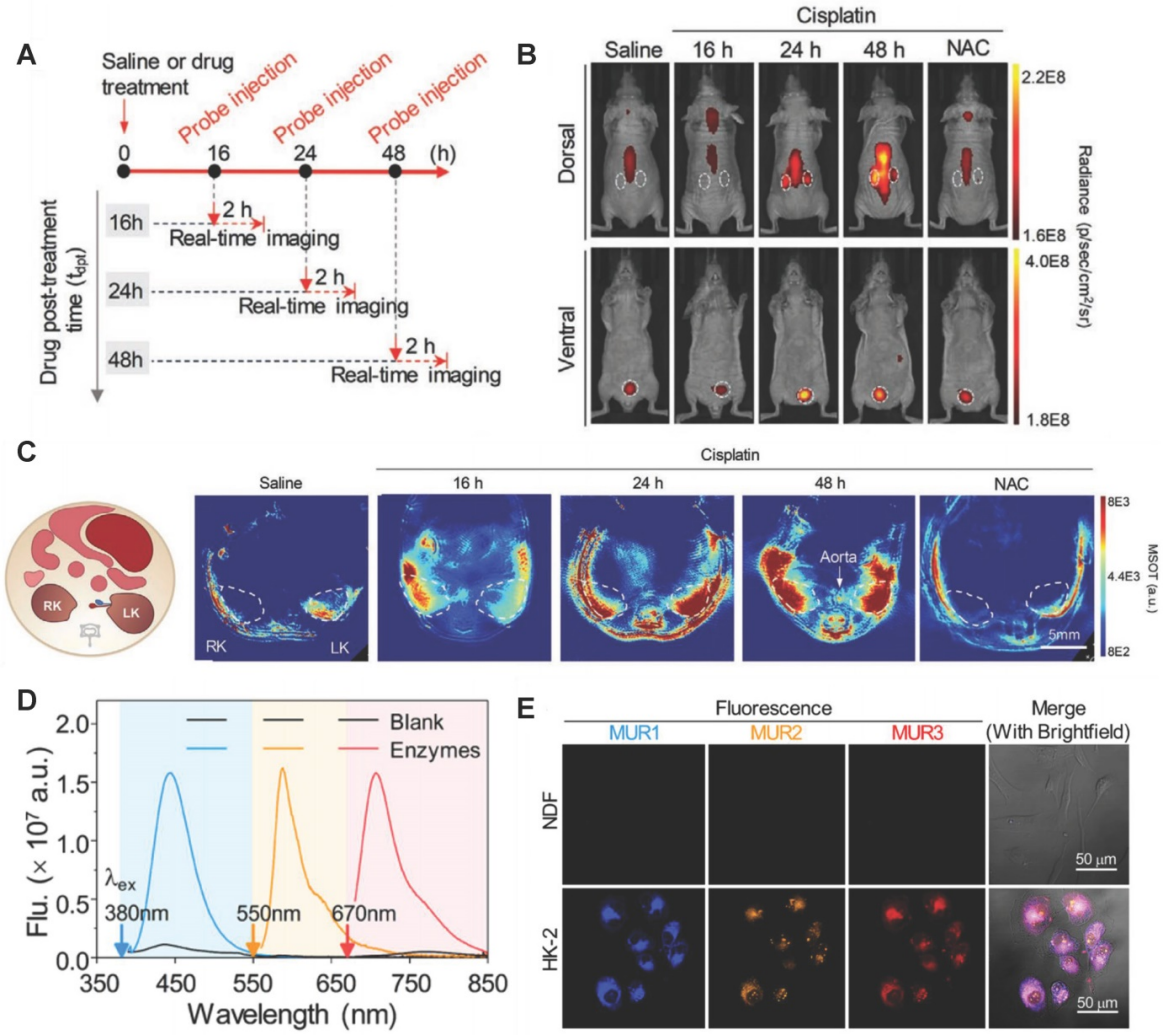
(A) Timeline for development of cisplatin-induced AKI and bimodal imaging. (B) Representative NIRF images of living mice 60 min after intravenous injection. (C) Representative PA images of mice transverse section at 120 min after i.v. injection of FPRR in different treatment groups (700 nm). Adapted with permission from 139, copyright 2020 (D) Fluorescence spectra of MURs cocktail in the absence or presence of all three biomarkers GGT, AAP, and NAG. (E) Multiplex fluorescence images of human primary dermalfibroblasts (NDF) and kidney proximal tubule epithelial cells (HK-2) after incubation with MUR1-3. Adapted with permission from 145, copyright 2020

GGT is a renal tubular brush border enzyme anchored on the outer surface of the cytoplasmic membrane, and the concentration of GGT in human serum is less than 50μm/L under normal physiological conditions. Many GGTs are released into the urine or blood from the damaged renal tubular epithelial cells through exocytosis or leakage when AKI occurred 136. The concentration of GGTs raises earlier than SCR and UO. Therefore, GGT is adopted as early diagnostic biomarker of AKI. For example, Zhou *et al.* found GGT increased the accuracy of AKI prediction of postoperative AKI in patients with hepatocellular carcinoma 137. Rethinam *et al.* also used GGT as an indicator of AKI in the renal protective effect of sphaeranthus amaranthoides 138. Traditional GGT imaging methods cannot accurately diagnose AKI early due to lack of kidney specificity. Currently, GGT is detected with high specificity by fluorescence method through the specific enzyme catalytic properties of GGT. For example, Cheng *et al.* developed a fluoro-photoacoustic polymeric renal reporter (FPRR) for detecting GGT 139. FPRR consisted of three parts: renal scavenger (dextran), NIRF/PA signal substance (CyOH) and GGT reaction site (γ-glutamate). The hydroxyl group of CyOH was connected to γ-glutamic acid through p-aminobenzyl alcohol to weaken the electron donating ability of oxygen atoms. The amide bond of γ-glutamic acid was specifically cleaved by GGT to generate dextran instead of CyOH (Dex-CD) with strong fluorescence and PA signal. FPRR was very sensitive to the detection of GGT. GGT increased the NIRF signal by 33 times and the PA signal by 6 times of the FPRR detection system. More importantly, FPRR detected AKI 48 hours earlier compared with creatinine detection in cisplatin-induced AKI mouse model (**Figure 11A-C**). And FPRR cytotoxicity test by MTS assay in HK-2 and NDF living cells confirmed its low cytotoxicity.

GGT concentration also increases in the early stages of hepatobiliary disease and certain tumors 140-144. Testing GGT alone leads to false positive results to diagnose AKI. Recently, Cheng *et al.* developed a testing strategy to improve the accuracy of early diagnosis of AKI for simultaneously detecting multiple interrelated biomarkers with a multiple optical analysis system MUR1-3 145. MUR1-3 contained linking groups that were specifically cleaved by GGT, alanine aminopeptidase (AAP) and NAG, respectively. AAP was also an enzyme released from damaged tubular cells upon AKI and was often used as an indicator of renal function evaluation 146. MUR1 was composed of GGT response group L-glutamic acid γ-(7-amino-4-methylcoumarin) for GGT, MUR2 had an alanine linker for AAP, and MUR3 had the N-acetyl-β-glucosamine for NAG. MUR1-3 had weak fluorescence in their inherent state. After being activated by GGT, AAP and NAG, their blue, orange, and near-infrared fluorescence signals with small spectral overlap were increased dozens of times (**Figure 11D-E**). MUR1-3 diagnosed AKI 48 hours earlier than current clinical diagnostic methods, and had better diagnostic accuracy in the cisplatin-induced AKI mouse model.

## 6. KIM-1

KIM-1 is a potential early biomarker of AKI because KIM-1 is almost not expressed in healthy kidney epithelial cells 147-149. KIM-1is highly expressed in proximal tubule cells 2 hours after AKI. The extracellular domain of KIM-1 formed a 90kDa soluble protein under the hydrolysis of metalloproteinases during AKI. Elevated KIM-1 levels correlated with a decline in eGFR in a study including 4750 patients followed for more than 10 years 150. Manuel J *et al.* also found KIM-1 in patients with COVID-19 might provide additional value in recognizing AKI at an early stage of disease in a cohort of 80 patients with COVID-19 151. However, AKI is serious when the concentration of KIM-1 in blood or urine increased. Therefore, the traditional ELISA method of detecting KIM-1 in serum or urine can't provide early diagnosis of AKI.

**Figure 12:**
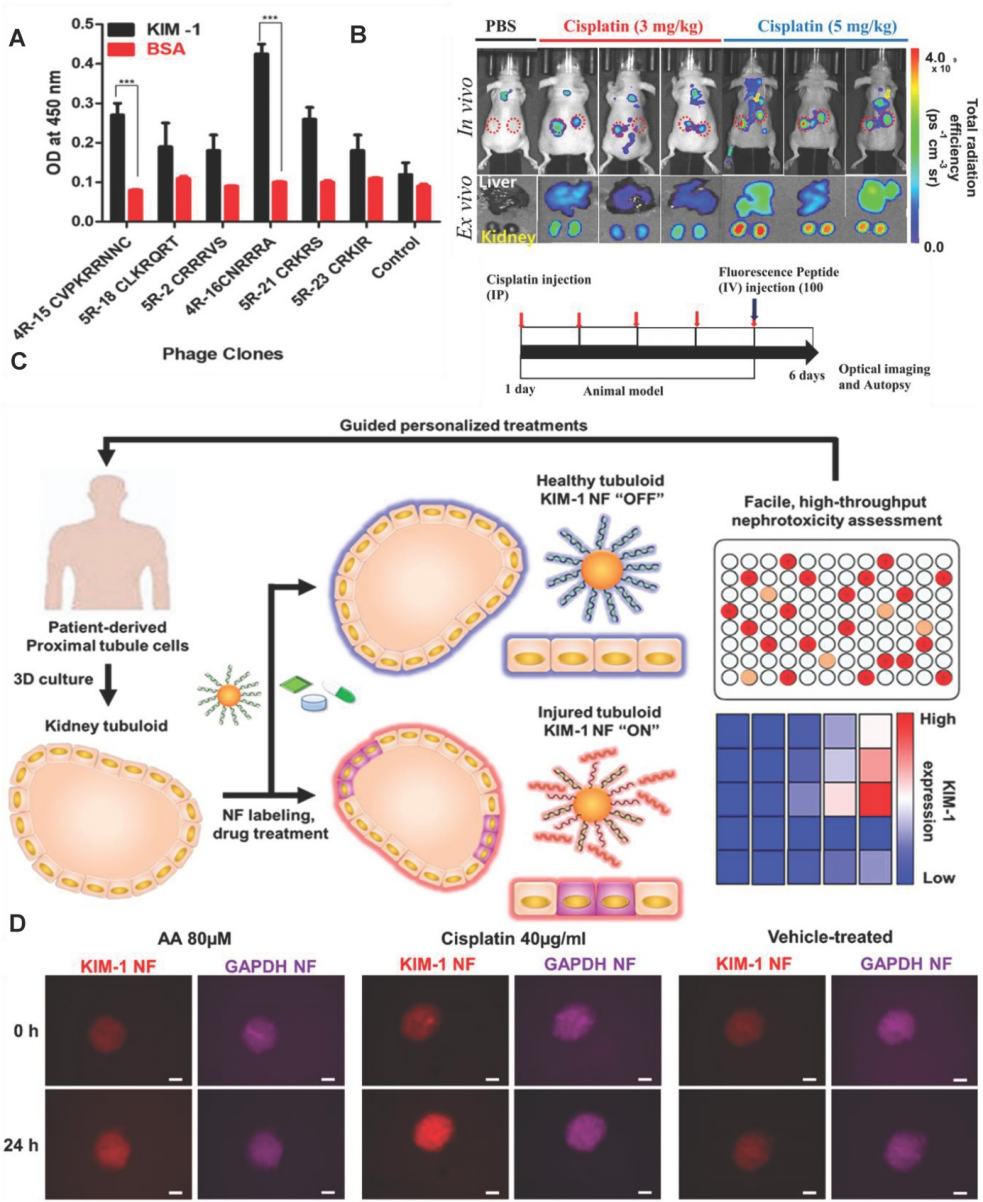
(A) Peptide-displaying phage clones selected through biopanning bind selectively to the recombinant KIM-1 protein. The binding efficiencies of the peptide-displaying phage clones (selected through biopanning) with KIM-1 recombinant protein were determined using ELISA. (B) *In vivo* imaging for the detection of drug-induced kidney damage using the labeled CNRRRA peptide. Adapted with permission from 152, copyright 2021 (C) Schematic illustration showing KIM-1 NF-assisted nephrotoxicity assessment. (D) Representative images showing aristolochic acid-treated, cisplatin-treated, and vehicle-treated tubuloids. Adapted with permission from 153, copyright 2021

Recently, Kwon *et al.* developed an *in vivo* KIM-1 NIF imaging probe for early diagnosis of AKI 152. The KIM-1 probe was composed of fluorescent molecule Flamma675 and the peptide CNRRRA with high affinity for KIM-1 (**Figure 12A**). The probe specifically recognized KIM-1 to emit NIF at proximal tubule cells in the damaged kidney tissue. The probe effectively imaged the kidney tissue two hours after the kidney injury (**Figure 12B**). In addition to KIM-1, KIM-1 mRNA is also an effective biomarker for early diagnosis of AKI. Recently, Wiraja *et al.* reported a gold nanoflare (NF) sensor with oligonucleotide modification to monitor KIM-1 mRNA 153 (**Figure 12C**). The probe adopted NFs as an efficient fluorescence extinguishing agent. The surface of NFs was connected by Au-S bonds to a DNA double-strand consisting of a long strand with the complementary sequence of KIM-1 mRNA and a short strand labeled with a fluorophore. KIM-1 mRNA promoted the release of short-stranded DNA from the surface of NF by pairing with long-stranded DNA to restore fluorescence. KIM-1NF effectively detected renal tubular damage induced by nephrotoxic drugs 24-72 hours later. The probe showed strong fluorescence in cisplatin-induced nephrotoxic renal tubular epithelial cells (**Figure 12D**). The increase of KIM-1 mRNA expression in in renal tubular cells should be earlier than the increase of KIM-1 expression. Therefore, the probe theoretically had great potential for early diagnosis of AKI.

## 7. miRNA-21

**Figure 13:**
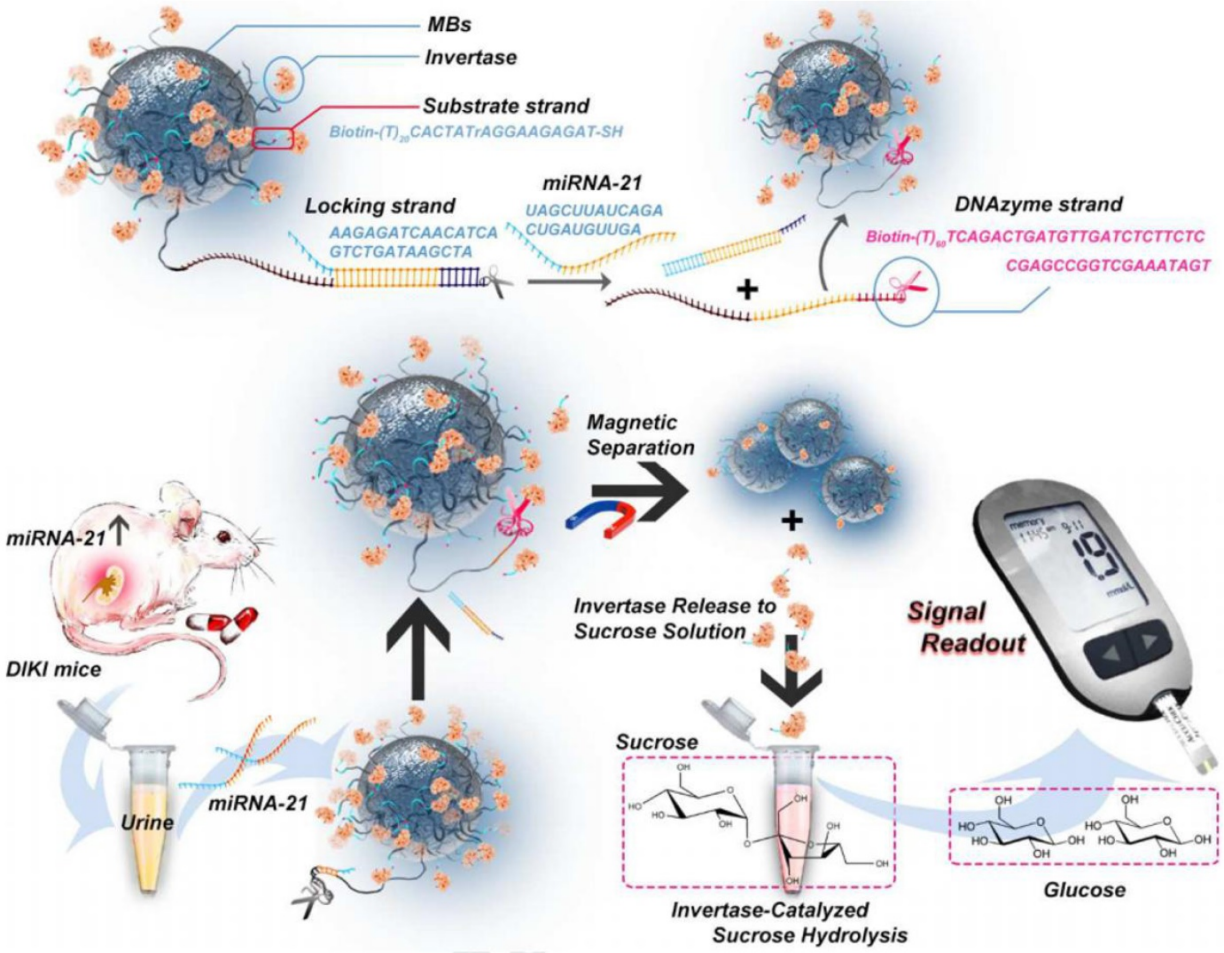
Schematic diagram of constitution of MBs-DNA-Inv and mechanism for the detection of miRNA-21 based on PGM and dual signal amplification. Adapted with permission from 168, copyright 2020

miRNAs are a type of short endogenous non-coding molecules with about 18-25 nucleotides 154. The dysregulation of miRNAs is closely related to the pathophysiological process of AKI 155-165. For example, the level of miRNA-21 in AKI patients is more than five times higher than that in healthy persons. miRNA-21 has a protective effect on renal ischemia-reperfusion injury by regulating the PDCD4, PTEN and Akt apoptosis signaling pathways. Therefore, miRNA-21 has been adopted as a biomarker for early AKI 166. Young *et al.* found that urinary exosomal miRNA-21 level was higher in the AKI group than in the non-AKI group and the miRNA-21 level correlated inversely with the estimated glomerular filtration rate, which demonstrated miRNA-2 was a surrogate biomarker for scrub typhus-associated AKI diagnosis 167. The traditional detection methods of miRNA-21 like quantitative reverse transcription polymerase chain reaction (qRT-PCR) lacke the convenience and effectiveness. Recently, Huang *et al.* proposed an instant detection method for miRNA-21 based on a personal blood glucose meter (PGM) 168 (**Figure 13**). As a widely used personal diagnostic equipment, PGM was lightweight, easy to operate, and reliable for quantitative detection 169. A dual signal amplification strategy of invertase and RNA cleaving DNA enzyme was adopted to improve detection sensitivity of miRNA-21. Magnetic beads (MB) were functionalized with two DNA double strands: a substrate chain linked with invertase and a locking double strand. The locking double strands were composed of a single strand with DNA enzyme function (DNase) and its complementary paired strands. miRNA-21 hybridized with the complementary strand of DNase and released the DNase single strand to cut the substrate chain in the presence of Mn^2+^. In this case, the invertases were detached from the MB. After magnetic separation of the undetached invertase, the invertases converted sucrose into glucose which was detected by the PGM. Thanks to the dual-enzyme amplification effect and the convenience of PGM, the method efficiently detected miRNA-21 in urine samples with a detection limit as low as 68.08fM/L.

In order to further improve the detection sensitivity and stability, Xu et al. developed an electrochemiluminescence biosensor for miRNA-21 by signal amplification technology with targeted induction hybridization chain reaction (HCR) 170. HCR didn't require the participation of enzymes and complicated temperature changing apparatus, and was an effective and stable signal enhancement strategy 171. The magnetic bead capture probe complex (SAMBs-CPH1) was formed by the combination of streptavidin-modified magnetic beads (SAMBs) and biotin-modified capture probe H1 (CPH1). miRNA-21 specifically opened the neck loop structure of CPH1 through base complementary pairing to expose its sticky ends to continuous synthesis Double-stranded DNA (dsDNA) by triggering the HCR. Tris(1,10-phenanthroline) ruthenium (II) chloride hydrate (Ru(Phen)_3_^2+^) was further inserted into the groove of dsDNA to form dsDNA-Ru(Phen)_3_^2+^ to enhance electrochemiluminescence signal. The electrochemiluminescence sensor detected miRNA-21 in the urine of AKI patients with high sensitivity, stability, and accuracy, with a detection limit of 0.14fM/L.

## 8. Summary and Prospects

In this review, we summarize the latest advances of imaging probes, biosensors and machine learning to diagnose early AKI. These methods showed a superior prospect for AKI early diagnosis due to many unique advantages, including deep penetration, high speed, good biocompatibility, high renal clearance rate, high accuracy and so on. However, these methods still face some challenges for early diagnosis of AKI in clinical application.

First, the machine learning models for AKI prediction have made a lot of progress, but there are still some problems to be solved. The classification criteria of AKI have a greater impact on the clinical prediction results. However, these models vary in their classification criteria for AKI and mostly don't consider UO criteria, potentially rendering some cases missed. And kinds of indicators are hard to be quantified, for example, mental status of patients and the color of mucous. Further, the databases established by many models come from a single medical center, which easily overfit and lead to poor cross-site transportability. In addition, many organizations prefer to perform model on site to avoid leaking sensitive information to others due to privacy concerns. Therefore, the application of these models also requires powerful real-time computing capabilities.

Secondly, the optical and PA imaging probes of RONS are still in the laboratory research stage. These probes adopt NIR as the excitation light source and have a deeper penetrating ability than visible light, which are highly effective in early diagnosis of AKI in mouse models. However, there are huge species differences between mice and humans. For example, the body weight of humans is much heavier than that of mice. The kidney is generally located about 10-20 cm deep in the skin of a human body, while the mouse is usually only 0.5-1 cm. Therefore, these imaging probes face big problems of insufficient penetration and weak signal when transplant into clinical applications. Here, a new type of kidney RONS response imaging probe with the deeper penetration capability is the future development direction, such as RONS-responsive magnetic resonance imaging and positron emission tomography imaging probes.

Third, the detection of early AKI specific biomarkers such as NGAL, KIM-1 and miRNA-21 in blood or urine don't have the problem of tissue penetration. These methods are easier to translate into clinical applications. However, these biomarkers are usually only a few hours earlier than the detection of SCR and UO, which not only put forward very stringent requirements for detection, but also make the intervention time window for AKI very short. In addition, some biomarkers also exist in other diseases and cause false positives. Therefore, the development of more rapid and accurate detection methods is the future direction. In addition, it is necessary to conduct an in-depth study of the early pathological characteristics of AKI to find new earlier and more specific biomarkers.

Finally, we believe that more and more accurate methods for diagnosing early AKI will be developed and translated into clinical applications with the deepening of interdisciplinary research. These accurate methods for early diagnosis of AKI can be very effective in reducing the incidence and mortality of AKI.

## Figures and Tables

**Table 1:** Machine learning related concepts involved in this article

Concepts	Introduction
Linear regression	A simple algorithm for regression task which expects a hyperplane to fit the dataset (a straight line when there are only two variables) 36
Generalized additive model	A method of constructing a non-monotonic response model within the framework of a linear or logistic regression model (or any other generalized linear model) 37
Decision Tree	A simple but widely used classifier for classifying unknown data by building decision trees from training data 38
Support vector machine	Transform classification problem into the problem of finding the classification plane and the classification is achieved by maximizing the distance between the boundary points of the classification and the classification plane 39
Logistic regression	Deal with binary classification problems where the dependent variable is a categorical variable 40
Gradient boosting decison tree	The purpose is to learn a series of weak classifiers or basic classifiers from the training data, and then combine them into a strong classifier 41
Neural networks	Abstract the human brain neuron network from the perspective of information processing, establish a certain simple model, and form different networks according to different connection methods 42
Random forest	Integrated algorithm composed of many decision trees 38
Genetic algorithm	A computational model of searching for the optimal solution by simulating the natural selection and genetic mechanism of Darwin's biological evolution theory 43
Deep Taylor decomposition	A method to explain the prediction results of the neural network to the individual; The result it produces is the decomposition of the function expressed by the neural network on the input variables 44
Principal component analysis	Analyze the data to identify patterns and find patterns to reduce the dimensionality of the data set while minimizing information loss 45
Shap	An interpretation technique based on the Shap value of each feature; A positive Shap value indicates that the feature causes a higher risk of disease, while a negative Shap value is the opposite 46

**Table 2:** Methods of machine learning to predict AKI

Category	Modeling method	Dataset source	Limitations	Optimal AUC	Refs
Preoperative AKI Risk Prediction	RF	The University of Florida Health Integrated Data Repository	Single-center study; no clear definition of features	0.88	47
	LR	The hospital database, electronic records, chart review and the catheterization reports in the Erasmus Medical Center	Single-center study; urine output was not considered when defining AKI.	0.79	49
AKI Prediction During Surgery	SVM,LR, RF,GBDT, DNN	The preoperative assessment record, anesthesia record and EHR	Single-center study; ignoring some key features	0.85	50
	LR, Decision Tree,SVM,RF,GBDT	Electronic medical records and records on intraoperative variables at Far Eastern Memorial Hospital	Single-center research; manual input of features; data imbalance	0.84	51
	DNN	Perioperative Data Warehouse	Single-center study; loss of creatinine value caused lots of cases to be lost.	0.792	52
Postoperative AKI Real-time Prediction	RNN	EHR at a tertiary care center for cardiovascular diseases	The observation period for patients varies in length.	0.90	56
Intensive Care Unit AKI Prediction	RF	The Multidisciplinary Epidemiology and Translational Research in Intensive Care Data Mart	Unbalanced data sources; AKI was not manually reviewed; Incomplete AKI definition	0.88	57
	RF	The EPaNIC multicenter randomized clinical trial database	NGAL is only measured in the verification queue	0.84	58
	Integrated classification learning	the PICU and CTICU of three independent tertiary-care pediatric intensive care centers	Urine volume standards were not considered when defining AKI; patients with uremia were not excluded.	0.89	59
AKI Prediction in All Hospital Wards	RNN	The U.S. Department of Veterans Affairs clinical database	representative cases are uneven	0.92	60
	GBDT	The Clinical Research Data Warehouse at the University of Chicago	Urine volume standards were not considered in the definition of AKI; baseline SCR was inaccurate	0.90	61
	LR	The Yale-New Haven Health System	The drug dose is not considered in the drug variables.	0.81	62
Interpretable AKI Prediction Model	TCN	HER of all residents of four Danish municipalities	the definition of AKI need improvement	0.88	66
Cross-site Transportability Model for AKI Prediction	GBDT	EHR data from a source healthcare system	baseline SCR is inaccurate;miss the key variables of heart rate, blood oxygen saturation and Braden scale score	0.92	67

**Table 3:** Methods of biomarkers detection for early diagnosis of AKI

Category	Probe/Method name	Target biomarker	Advantages	Refs
Near-infrared fluorescence imaging probe	NIR-O_2_^.-^	O_2_^.-^	It is the first near-infrared fluorescent O_2_^.-^ probe in AKI detection.	172
	MRP1-3	caspase-3, NAG,O_2_^.-^	HPβCD enhance its renal clearance rate	93
	TA-TPABQ	H_2_O_2_	raw nano-material enhance its renal clearance rate	94
	KNP-1	ONOO^-^	good renal targeting	95
	KTP5-ICG-GNP	ROS	Realize long-term monitoring of renal dysfunction	99
	Naph-O_2_^.-^	O_2_^.-^	Imaging depth up to 130μm	172
	MUR1-3	GGT,AAP,NAG	multiple optical analysis improve accuracy	145
Chemiluminescence Imaging Probe	MRPD	O_2_^.-^	Dual channel detection is more reliable	93
	NCR1	O_2_^.-^	Higher resolution and less optical signal loss	106
	NCR2	ONOO^-^	Higher resolution and less optical signal loss	106
Photoacoustic Molecular Imaging Probe	FDOCl-22	HOCl	high renal clearance rate and deep imaging depth	108
	SiRho-HD	ONOO^-^	self-calibrate and eliminate interference.	110
	FPRR	GGT	High imaging depth	139
Electrochemical immunosensor	Peptide-mediated sensor	NGAL	Good stability and short analysis time	127
	Aptamer-mediated sensor	NGAL	Good stability and short analysis time; reusable	129
	Homogeneous electrochemiluminescence biosensor	miRNA-21	high sensitivity	170
Surface plasmon resonance biosensor	Refreshable nanobiosensor	NGAL	Good stability, reusable	133
Surface enhanced raman spectroscopy	SERS specific immunoassay	NGAL	Different molecular forms of NGAL can be distinguished	135
Fluorescence immunoassay	Flamma675- CNRRRA	KIM-1	*In vivo* imaging, earlier diagnosis	152
Nanoflare sensor	Spherical nucleic acid-based mRNA nanoflares	KIM-1	Direct detection of mRNA, earlier diagnosis	153
Point-of-Care Testing	PGM	miRNA-21	Real-time monitoring, portable	168

## Abbreviations

AKI: acute kidney injury
RRT: renal replacement therapy
SCR: serum creatinine
UO: urine output
NGAL: neutrophil gelatinase associated lipoprotein
GGT: γ-glutamyl transpeptidase
KIM-1: kidney injury molecule-1
MiRNA: microRNA
RONS: excess reactive oxygen species and nitrogen
SPR: surface plasmon resonance
DNA: deoxyribonucleic acid
EHR: electronic health records
AUC: area under the curve
ROC: receiver operating characteristic curve
PAPI: pulmonary artery pulsatility index
RAP: right atrial pressure
LR: logistic regression
SVM: support vector machine
RF: random forest
GBDT: gradient boosting decision tree
DNN: deep neural network
XGBoost: extreme gradient boosting
OFS: original feature set
MAP: minimum map feature
RFS: simplified feature set
RNN: recurrent neural network
ICU: intensive care unit
TCN: temporal convolutional network
DTD: deep taylor decomposition
PRC: precision recall curve
HOCl: hypochlorous acid
NIF: near-infrared fluorescence
PA: photoacoustic
CyOH: cyanine fluorescent dye
HPβCD: 2-hydroxypropyl)-β-cyclodextrin
TA: tannic acid
ICG: indocyanine green
GFR: glomerular filtration rate
HD: homodimer dyes
FRET: förster resonance energy transfer
AAP: alanine aminopeptidase
PGM: personal blood glucose meter
MB: magnetic beads
DNase: DNA enzyme
HCR: hybridization chain reaction
SERS: surface enhanced raman spectroscopy
